# *Leishmania major* surface components and DKK1 signalling via LRP6 promote migration and longevity of neutrophils in the infection site

**DOI:** 10.3389/fimmu.2024.1473133

**Published:** 2024-10-22

**Authors:** Olivia C. Ihedioha, Haley Q. Marcarian, Anutr Sivakoses, Stephen M. Beverley, Diane McMahon-Pratt, Alfred L. M. Bothwell

**Affiliations:** ^1^ Department of Pathology, Microbiology, and Immunology, University of Nebraska Medical Center, Omaha, NE, United States; ^2^ Department of Molecular Microbiology, Washington University School of Medicine in St Louis, St. Louis, MO, United States; ^3^ Department of Epidemiology of Infectious Diseases, Yale School of Public Health, New Haven, CT, United States

**Keywords:** leishmaniasis, neutrophils, apoptosis, platelet, innate response

## Abstract

**Background:**

Host-related factors highly regulate the increased circulation of neutrophils during *Leishmania* infection. Platelet-derived Dickkopf-1 (DKK1) is established as a high-affinity ligand to LRP6. Recently, we demonstrated that DKK1 upregulates leukocyte-platelet aggregation, infiltration of neutrophils to the draining lymph node and Th2 differentiation during *Leishmania* infection, suggesting the potential involvement of the DKK1-LRP6 signalling pathway in neutrophil migration in infectious diseases.

**Results:**

In this study, we further explored the potential role of DKK1-LRP6 signalling in the migration and longevity of activated neutrophils in the infection site using BALB/c mice with PMNs deficient in LRP6 (LRP6^NKO^) or BALB/c mice deficient in both PMN LRP6 and platelet DKK1 (LRP6^NKO^ DKK1^PKO^). Relative to the infected wild-type BALB/c mice, reduced neutrophil activation at the infection site of LRP6^NKO^ or LRP6^NKO^ DKK1^PKO^ mice was noted. The neutrophils obtained from either infected LRP6^NKO^ or LRP6^NKO^ DKK1^PKO^ mice additionally showed a high level of apoptosis. Notably, the level of LRP6 expressing neutrophils was elevated in infected BALB/c mice. Relative to infected BALB/c mice, a significant reduction in parasite load was observed in both LRP6^NKO^ and LRP6^NKO^ DKK1^PKO^ infected mice. Notably, DKK1 levels were comparable in the LRP6^NKO^ and BALB/c mice in response to infection, indicating that PMN activation is the major pathway for DKK1 in promoting parasitemia. Parasite-specific components also play a crucial role in modulating neutrophil circulation in *Leishmania* disease. Thus, we further determine the contribution of *Leishmania* membrane components in the migration of neutrophils to the infection site using null mutants deficient in LPG synthesis (*Δlpg1^-^*) or lacking all ether phospholipids (plasmalogens, LPG, and GIPLs) synthesis (*Δads1^-^*). Relative to the WT controls, *Δads1^-^* parasite-infected mice showed a sustained decrease in neutrophils and neutrophil-platelet aggregates (for at least 14 days PI), while neutrophils returned to normal in *Δlpg1^-^* parasite-infected mice after day 3 PI.

**Conclusion:**

Our results suggest that DKK1 signalling and *Leishmania* pathogen-associated molecular patterns appear to regulate the migration and sustenance of viable activated neutrophils in the infection site resulting in chronic type 2 cell-mediated inflammation.

## Graphical Abstract

Created in BioRender. Ihedioha, O. (2024) BioRender.com/h48c664.

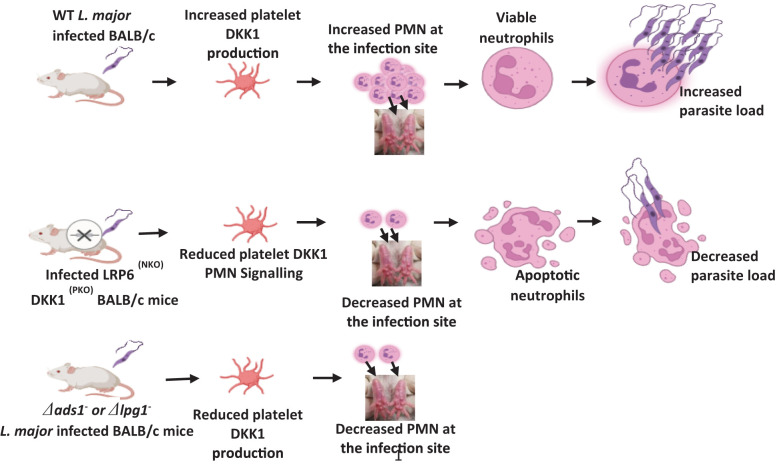

## Introduction

The intracellular protozoan *Leishmania major (L. major)* is an obligate parasite of macrophages and other phagocytic cells that can infect various mammalian hosts, such as rodents, dogs, and humans. In the Old World, cutaneous leishmaniasis is mainly caused by *L. major*, and in humans, it is a self-limiting persistent infectious disease; the murine model has proven useful in dissecting the immune response to infection (1). In the 1980s, resistance and susceptibility to infection with *L. major* was correlated with the sustained activation of parasite-reactive CD4^+^ Th1 or Th2 cells, respectively (2). Recent studies reported that susceptibility to *L. major* might not be due to a Th2 bias but as a result of an imbalance in the number of IL-10 and IL-4-producing regulatory T cells that are activated during infection in BALB/c mice (3, 4).

Events involving neutrophils occurring during the first days following infection are important in innate immunity and instructing subsequent Th differentiation (5–7). Polymorphonuclear leukocytes are professional phagocytes present within the blood during steady-state conditions (8). Once the steady state of a peripheral tissue is disrupted, neutrophils are the first cells recruited within the tissues (8). In response to *Leishmania* infection, activated neutrophils are rapidly recruited to the infection site following the delivery of parasites by sand fly bite or needle injection (9, 10). Local inflammatory signals induced by infection attract neutrophils that infiltrate the inoculation site through the vascular endothelium (11–13). Naïve neutrophils are inherently short-lived cells with a half-life of only ∼6–19 h in circulation, after which they undergo apoptosis (14, 15). *Leishmania* can prolong the lifetime of neutrophils to secure an intracellular environment for its survival (16). Thus, PMNs can play an unfavorable role in the development of leishmaniasis. This is confirmed by previous studies that showed early influx of neutrophils instructs and promotes the induction of Th2 responses and susceptibility to *L. major* infection (9).

The components of the *Leishmania* surface coat are densely packed with glycosylphosphatidylinositol (GPI)-anchored glycoconjugates, which are essential determinants in intracellular parasitic survival, replication, virulence and pathogenesis in the insect vector and mammalian host (17–19). These GPI-anchored molecules include lipophosphoglycan (LPG, containing 15-30 copies of a phosphoglycan repeating unit), GPI-anchored proteins (membrane proteophosphoglycans, gp63 and gp46), and a heterogeneous group of small glycosylinositolphospholipids (GIPLs) (18, 20). A shared structural motif of GPI-anchored glycoconjugates and other abundant phospholipids in *Leishmania* is the presence of ether lipids (20). Ether phospholipids are major *Leishmania* membrane components that occur separately or as part of the GPI-anchored molecules implicated in virulence (20). The GPI anchor of LPG has similarities with those present in both GIPLs and GPI-anchored proteins (21). Typically, studies to understand the role of LPG were done using purified LPG (22). However, the similarity of LPG domains to those of other parasite molecules raises the issue of specificity and the possibility of cross-activity. For instance, numerous LPG functions have also been assigned to GIPLs and other GPI-anchored proteins (23–26). Teasing out the specific contributions of either LPG, GIPLs or ether phospholipids within the complex milieu of the parasite glycocalyx remains a major challenge. This can be overcome in part using *L. major* genetically deficient in only LPG or a combination of LPG, GIPLs and ether phospholipids. Biochemical studies showed that *Δads1* null mutant parasites deficient in LPG, GIPLs and ether phospholipids (27) synthesize a normal amount of GPI-anchored proteins (e.g. gp46) (20). Likewise, the *LPG1* mutant was generated by targeted inactivation of a putative galactofuranosyltransferase required for the synthesis of the LPG glycan core; the *LPG1* mutant expressed normal levels of PPG, GPI anchored proteins and GIPLs (28). The GPI anchors of GIPLs and LPG (with its long-chain alkyl group) have been demonstrated to suppress host cell responses (inhibition of nitric oxide production and protein kinase C) (20, 26, 29, 30).

Dickkopf-1 (DKK1), initially identified in *Xenopus*, is an inhibitor of β-catenin-dependent Wnt signaling (31, 32). The mechanism of DKK1 blocking the Wnt signalling is demonstrated by its competitive binding to the low-density lipoprotein receptors (LRP5 and 6 complexes) with markedly higher affinity than its counterpart agonist Wnt3a (31, 33). Previous studies have reported that circulating levels of DKK1 are associated with numerous inflammatory diseases, and LRP6-DKK1 interaction is required for the regulation of inflammation and cell proliferation (34, 35).

Our previous work has shown the importance of LPG-induced DKK1 in driving Th2 immune response, leukocyte platelet aggregate formation, and migration of neutrophils to the draining lymph node during *L. major* infection (31). This suggests that the interaction of DKK1 and its receptor (LRP6) regulates inflammatory response and the outcome of *Leishmania* infection. In this study, we explored the role of DKK1 and LPR6 signalling in the migration and maintenance of viable activated neutrophils at the site of infection during the early development and establishment of disease (days 3 to 14). This was achieved by conditional deletion of LRP6 in neutrophils and DKK1 from platelets. Furthermore, we examined whether parasite surface components are required for continual neutrophil activation and migration to the infection site at the later stage of infection. Overall, this study suggests that local inflammatory signals induced by *Leishmania* PAMPs and DKK1-LRP6 interaction contribute to the neutrophil full effector function at the infection site and differentiation of Th2 cytokines.

## Experimental procedures

### Mice

Previous studies demonstrated the expression of LRP5 in murine neutrophils (36). However, LRP5 and 6 have slightly different modes of action, they are not always expressed or paired together (37). It is not clear whether murine neutrophils express LRP6. Thus, to determine the neutrophil expression of LRP6, PMN cells were harvested from the bone marrow of non-infected BALB/c mice (as described below) and examined for LRP6 expression using unconjugated rabbit anti-LRP6 polyclonal antibody (Thermo Fisher Scientific- BS-5408R) for 30 mins in the dark at room temperature before staining with Alexa Fluor 647-conjugated secondary anti-rabbit IgG antibody (Invitrogen). Results clearly indicated the expression of LRP6 on a significant subpopulation of neutrophils (**Supplementary Figure S1**). Consequently, we sought to examine the role of LRP6 on PMNs using genetically deficient mice.

WT BALB/c mice (4 weeks old) were purchased from the Jackson Laboratory. Sperm from mice heterozygous for LRP6 flox and MRP8-Cre recombinase was used for *in vitro* fertilization, and pups carrying a heterozygous floxed allele of LRP6 and MRP8-Cre were backcrossed to female BALB/c mice for eight generations. Thereafter, the heterozygous LRP6 floxed mice were intercrossed with mice expressing Cre recombinase under the control of the MRP8 promoter to generate mice in which LRP6 were selectively deficient in neutrophils (LRP6^NKO^). PF4-Cre-DKK1 deficient mice generated by specific deletion of DKK1 in megakaryocytes and platelets were crossed with LRP6 floxed mice to generate DKK1-LRP6 deficient mice (LRP6^NKO^ DKK1^PKO^). Genotyping of all the knockout mice was performed through standard PCR procedures (31, 38). All experiments were carried out with age-matched littermates unless specified otherwise, and all mice were housed at the University of Arizona Animal Care Facilities. The mouse protocols were approved by the University of Arizona in accordance with the Association for Assessment and Accreditation of Laboratory Animal Care International (AAALAC). Mice strains were transferred to the University of Nebraska Medical Center (UNMC) and utilized under approved protocols.

### Parasite strains and infection protocol

The *L. major* mutants (*Δlpg1* ^-^ and *Δads1* ^-^) were derivatives of WT *L. major* LV39 clone 5 and Friedlin background, respectively. The homozygous mutant lines were made by homologous gene replacement and maintained in selective media as described previously (28, 39–41). *Leishmania major* parasites were maintained at 26°C in M199 culture medium (Thermo Fisher Scientific) supplemented with 20% heat-inactivated FBS (Thermo Fisher Scientific), 20 mM HEPES (Sigma-Aldrich) and 50 ug/ml gentamycin (Thermo Fisher Scientific). Prior to using parasites for infection, metacyclic promastigotes were isolated from stationary-phase cultures using density gradient centrifugation (42, 43). Metacyclic promastigotes were washed three times in cold phosphate-buffered saline (PBS-Thermo Fisher Scientific) by centrifugation, resuspended in PBS at 2X10^8^/ml and 10 μl containing 2x10^6^ metacyclic promastigotes were injected intradermally into the top of the right hind footpad.

### Infected footpad neutrophil isolation

Neutrophils were isolated from the footpad using an established protocol (44). Briefly, infected footpads were cut right above the ankle and deskinned. Crosswise cutting was done in small increments starting from the bottom of the foot towards the ankle and minced using toothed forceps. To obtain more cells in the non-infected mice, neutrophils were generated by injecting PBS into the two feet of each mouse, and the cells on both feet were harvested. Also, the number of mice in the non-infected group increased from 5 to 10 mice, and cells were pooled for the analysis. Cells were separated from tissues using a cell strainer (70um pore size- Thermo Fisher Scientific), centrifuged at 300xg for 7 minutes, and resuspended in FACS buffer for cell staining.

### Isolation, purification and culture of bone marrow-derived neutrophils with recombinant DKK1

Bone marrow-derived neutrophils were isolated and purified as previously described (45). Briefly, tibias and femurs were dissected from euthanized mice. The bone marrow contents were flushed and filtered through a 70-μm cell strainer using HBSS-EDTA. The cell suspension was centrifuged at 400 g for 10 min (room temperature) and resuspended in 1 ml HBSS–EDTA. Bone marrow-derived neutrophils were purified using a three-layer Percoll gradient (78%, 69%, and 52% Percoll) and centrifuged at 1500 g for 30 min without braking. The neutrophils from the 69%/78% interface were harvested.

Isolated neutrophils were cultured in 24-well plates (1x10^6^ cells/ml/well) using RPMI-1640 medium supplemented with 10% fetal bovine serum and 100 U/ml penicillin-streptomycin. Neutrophils in culture medium were treated with various concentrations of R & D Systems generated rDKK1(10, 30, 50 and 100 ng/ml) before incubation at 5% CO_2_, 37°C. Apoptotic neutrophils were determined at various time points (0-, 4-, and 24 hrs) post-treatment using flow cytometry, as described below.

### Neutrophil apoptosis

Apoptosis and viability of neutrophils (1x10^6^/ml) isolated from the footpads of infected and non-infected BALB/c, infected LRP6^NKO^and infected LRP6^NKO^ DKK1^PKO^ mice were assessed by measuring phosphatidylserine (PS) exposure using FITC-labeled annexin V as previously described (46). Briefly, cells were stained with Pac-blue conjugated Ly6G (BioLegend) for 20 minutes at room temperature. Cells were washed and labelled with annexin V-FITC (BioLegend) for 15 min in the dark at 37°C. Cellular membrane integrity was assessed using propidium iodide (PI) (BioLegend). Similar FITC Annexin V staining was done with bone marrow-derived neutrophils harvested at 0-, 4- and 24 hrs post-treatment with rDKK1. Stained samples were evaluated within 2 hours by LSR II flow cytometry and analyzed using FlowJo software. Neutrophils were identified by their forward and side scatter characteristics and Ly6G expression. The Annexin V positive subpopulation identified apoptotic cells, while Annexin V negative subpopulation identified live cells.

### CD11b and MHC class II positive neutrophils

Assessment of neutrophil activation (CD11b and MHC class II positive cells) was performed by minor modification of previously described methods (47–50). Briefly, neutrophils isolated from footpads of infected and non-infected BALB/c mice, infected LRP6^NKO^ DKK1^PKO^ and infected LRP6^NKO^ mice were stained with FITC conjugated Ly6G (BioLegend), Alexa Fluor 700 conjugated CD11b (eBioscience), and PE-conjugated MHC class II (eBioscience) antibodies for 15 mins in the dark at room temperature. Similar staining with FITC conjugated Ly6G and PECy7 conjugated CD11b was done with cells isolated from the footpad of BALB/c mice infected with either *Δlpg1* ^-^, *Δads1^–^* or WT-*L. major* parasites. Stained samples were evaluated within 2 hours by LSR II flow cytometry and analyzed using FlowJo software. Neutrophils were identified by their forward and side scatter characteristics and Ly6G expression. The CD11b and MHC class II positive subpopulation identified activated neutrophils.

### Myeloperoxidase positive neutrophils

Activated neutrophils were further determined by measuring myeloperoxidase (MPO) positive neutrophils using the established flow cytometry intracellular cell staining method with minor modifications (51). Briefly, cells (1x10^6^/ml) generated from the footpad of infected and non-infected BALB/c, infected LRP6^NKO^ and infected LRP6^NKO^ DKK1^PKO^ mice were fixed at room temperature in a tube with BD Cytofix™ Fixation Buffer (BD Biosciences) that has been prewarmed to 37°C. The cells were then washed and permeabilized with BD Perm/Wash™ Buffer (BD Biosciences). The permeabilized cells were stained in the dark at room temperature in BD Perm/Wash™ Buffer with FITC conjugated Ly6G and with either Alexa Fluor 647 mouse IgG1 isotype control or Alexa Fluor 647 conjugated myeloperoxidase antibody (BD Biosciences). Data acquisition and analysis were done using LSR II flow cytometry and FlowJo software, respectively.

### Neutrophil expression of LRP6 in infected mice

Neutrophils isolated from footpads of infected and non-infected BALB/c mice were stained with unconjugated rabbit anti-LRP6 polyclonal antibody (Thermo Fisher Scientific-BS-5408R) and Pacific-Blue conjugated Ly6G antibodies for 30 mins in the dark at room temperature before staining with Alexa Fluor 647-conjugated secondary anti-rabbit IgG antibody (Invitrogen). Data acquisition and analysis were done using LSR II flow cytometry and FlowJo software, respectively. Neutrophils were identified by their forward and side scatter characteristics and Ly6G expression.

### Neutrophil-platelet aggregation

Neutrophil-platelet aggregate assessment was performed as described previously with minor modifications (19). Briefly, cells (1x10^6^/ml) isolated from footpads of mice infected with *Δlpg1*-, *Δads1^-^* or WT-*L. major* parasites were stained with Pacific blue conjugated CD45 (BioLegend), FITC conjugated Ly6G (BioLegend), PE-conjugated CD41 (BioLegend), and APC conjugated CD11b (eBioscience) antibodies for 15 mins in the dark at room temperature. Samples were acquired by BD FACSCanto flow cytometry within 4 to 6 hours, and analysis was done using FlowJo software. Live gating was performed on leukocyte-sized events to exclude single platelets. Leukocytes were identified by their forward and side scatter characteristics and CD45 expression. The Ly6G+ and CD11b+ subpopulations identified activated neutrophils, while the Ly6G+ and CD41+ subpopulations were identified neutrophil platelet aggregates (NPA).

### DKK1 ELISA

The concentration of DKK1 in plasma was determined by Enzyme-linked immunosorbent assays using a mouse DKK1 ELISA kit (Thermo Fisher Scientific) according to the manufacturer’s protocol.

### Estimation of parasite burden

Parasite burden was estimated on day 14 post-infection by limiting dilution analysis as previously described (52).

### Statistical analyses

The *in vivo* percentage of neutrophils and NPA obtained from BALB/c mice infected with either *Δlpg1^-^*, *Δads1* ^-^ or WT-*L. major* was analyzed using one-way ANOVA with Bonferroni’s *post hoc* test. Comparison of neutrophil activation markers (MHC class II, CD11b and myeloperoxidase positive cells), MFI within the Ly6G+ cells and the number of neutrophils obtained from infected BALB/c, LRP6^NKO^, LRP6^NKO^ DKK1^PKO^ and non-infected BALB/c mice were done using one-way ANOVA with Bonferroni’s *post hoc* test. The mean fluorescent intensity (MFI) of CD11b and MHC II expressed by CD11b and MHC II positive/negative neutrophils were analyzed using Student’s t-test. Also, LRP6 expressed by neutrophils obtained from infected and non-infected BALB/c mice were analyzed using Student’s t-test. In addition, *in vitro* and ex vivo neutrophil apoptosis, viability, plasma DKK1 production and parasite burden were analyzed using one-way ANOVA with Bonferroni’s *post hoc* test. Data presented as mean +/- standard errors were performed using GraphPad Prism Software (GraphPad Software, San Diego, CA, USA).

## Results

### Reduced neutrophils and NPA formation in the infection site of *Δads1^-^* and *Δlpg1^-^* parasite-infected mice

**Figure 1:**
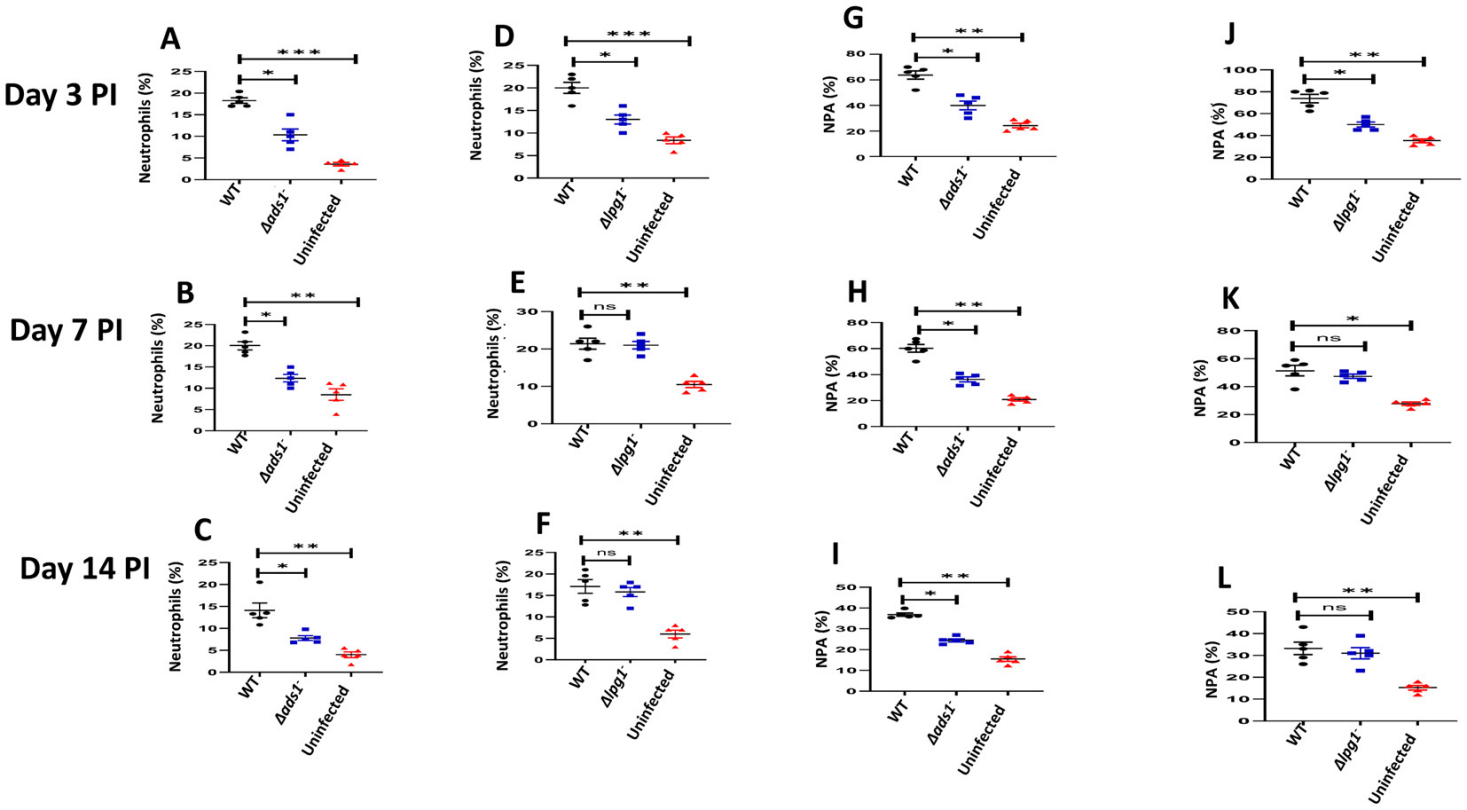
Decreased percentage of neutrophils and NPA in *Δads1^-^* and *Δlpg1^-^* parasite-infected mice. BALB/c mice were challenged with infective metacyclic promastigote (2 x 10^6^ parasites, n = 5) of (LV39c5 WT and *Δlpg1-*) and (Freidlin WT and *Δads1^-^*) strains via the footpad. Control mice (n = 10/2 feet per mouse) were given 0.9% NaCl saline. Neutrophils were isolated from the footpads of all infected and non-infected mice at days 3, 7 and 14 PI. Isolated neutrophil samples were analyzed by flow cytometry for Ly6G-positive cells and NPA formation. Each dot indicates Ly6G+ cells **(A–C)** and NPA **(G–I)** obtained from *Δads1* ^-^ parasite infected mice. Also, each dot indicates Ly6G+ cells **(D–F)** and NPA **(J–L)** obtained from *Δlpg1* ^-^ parasite infected Mice. Representative flow cytometry dot plots showing the analyses of Ly6G + cells and NPA performed on day 3 PI, as well as the concatenated dot plot of each sample in all the experimental groups are presented in **Supplementary Figure S2**. In all the experiments, WT-infected and non-infected mice served as positive and negative controls, respectively. Results are presented as mean (± SEM) and are representative of 3 independent experiments. One-way ANOVA with Bonferroni’s *post hoc* test was performed to analyze the data *p < 0.05, **p < 0.01, ***p < 0.001, ‘ns’ indicates not significant (p > 0.05).

The innate immune response to *Leishmania* begins with the recruitment of neutrophils to the infection site (53, 54). Given that recruitment of immune cells to the infection site is related to the expression of soluble chemotactic factors or signals released by cells in response to *Leishmania* parasites (53–55), we previously examined the possibility that *Leishmania* membrane components might play a role in neutrophil recruitment to the infection site and demonstrated that LPG-TLR2 (platelet) interaction was important to the initial (days 1-3) DKK1 release and PMN-Platelet aggregation. However, parasite-induced mechanisms related to subsequent DKK1 release were unclear as parasite LPG expression wanes with differentiation into the amastigote stage (56). To this end, BALB/c mice were infected with either *Δlpg1^-^*, *Δads1^-^* or WT-*L. major*, and the percentage of neutrophils at the infection site was determined at days 3, 7 and 14 PI. We found that the percentage of neutrophils in *Δads1^-^* parasite-infected mice at all the various time points was significantly reduced compared to the massive infiltrate seen in WT-infected mice. The *Δlpg1^-^* infected mice did show a significant decrease in the percentage of neutrophils at day 3 PI, which was restored at day 7 and 14 PI (**Figures 1A–F**). This is potentially related to LPG shutoff that occurs with amastigote transformation (56). Additionally, we have previously shown *in vitro* using soluble leishmania antigen (sLAG) from *L. major* parasites genetically deficient in LPG that DKK1 production is severely impaired. Further, LPA formation (*in vivo* studies) is significantly diminished for the parasites deficient in LPG. This suggests that the impact of surface LPG itself modulates cellular response and PMN recruitment.

The P-selectin expressed by activated platelets is a key to the formation of leukocyte platelet aggregates and effective leukocyte migration under inflammatory conditions (57). Platelet activation triggered by *L. major* is known to attract a subpopulation of effector monocytes to sites of *L. major* infection (58). To determine whether the parasitic components promote neutrophil platelet aggregate (NPA) formation, mice were infected with WT-*L. major*, *Δads1^-^* and *Δlpg1^-^* parasites, and NPA formation at the infection site was evaluated. Consistent with the decreased percentage of activated neutrophils at the infection site of *Δads1^-^* parasite-infected mice, data showed a reduced percentage of NPA in *Δads1^-^* parasite-infected mice (days 3, 7, and 14 PI) and *Δlpg1^-^* parasite-infected mice (day 3 PI) in comparison to WT-*L. major* infected mice (**Figures 1G–L**). Since *Δlpg1^-^* parasites lack LPG and *Δads1^-^* parasites are deficient in LPG, as well as GIPLs and ether phospholipids, these data suggest that these surface molecules might be critical to the continued migration of neutrophils and formation of NPA in the infection site. These results provide evidence that local inflammatory signals from *Leishmania* PAMPs may contribute to NPA formation and infiltration of neutrophils to the infection site.

### Impaired levels of CD11b and MHC class II positive neutrophils in LRP6^NKO^ and LRP6^NKO^ DKK1^PKO^ infected mice

**Figure 2:**
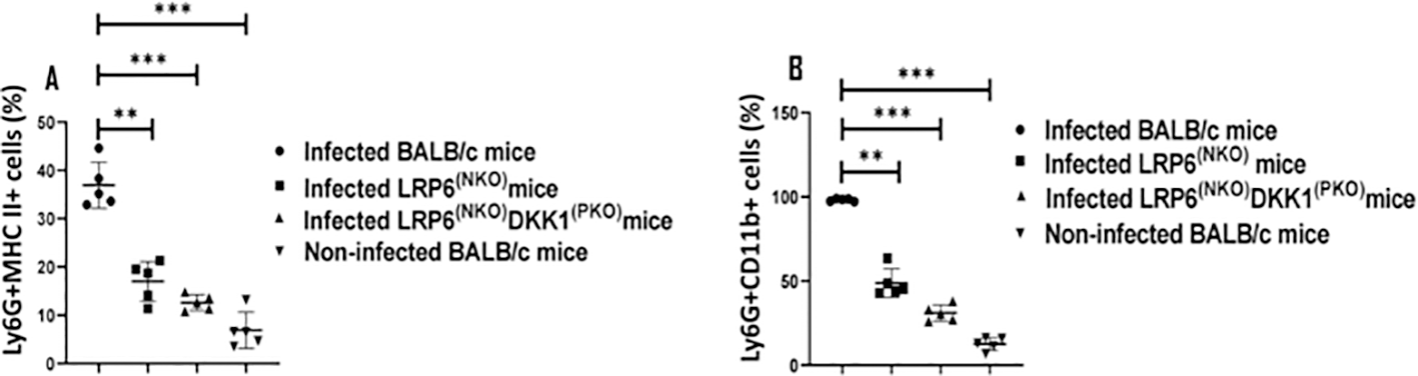
Decreased CD11b and MHC class II positive neutrophils derived from LRP6^NKO^ DKK1^PKO^ and LRP6^NKO^ infected mice. The WT-BALB/c, LRP6^NKO^and LRP6^NKO^ DKK1^PKO^ mice were challenged with infective metacyclic promastigote (2 x 10^6^ parasites, n = 5) of *L. major* via the footpad. Non-infected BALB/c mice (n = 10/2 feet per mouse) were given 0.9% NaCl saline. Neutrophils were isolated from the footpads of all infected and non-infected mice at day 3 PI. Isolated neutrophil samples were analyzed by flow cytometry for CD11b and MHC class II positive neutrophils. Each dot indicates MHC class II **(A)** and CD11b **(B)** positive cells. Representative flow cytometry dot plots showing the analyses of CD11b and MHC class II + neutrophils performed on day 3 PI, as well as the concatenated dot plot of each sample in all the experimental groups are presented in **Supplementary Figure S3**. In all the experiments, WT-infected and non-infected mice served as positive and negative controls, respectively. Results are presented as mean (± SEM). One-way ANOVA with Bonferroni’s *post hoc* test was performed to analyze the data **p < 0.01, ***p < 0.001(p > 0.05).

Neutrophil activation is commonly detected via increased CD11b and MHC class II expression levels in Ly-6G+ cells (59–62). We have shown that host-related factors such as DKK1 elevate LPA formation and trafficking of neutrophils to the draining lymph node under inflammatory conditions (31). This suggests that DKK1 signalling via its receptor (LRP6) might also regulate the migration of activated neutrophils to the infection site. Thus, to characterize whether DKK1 promotes infiltration of activated neutrophils to the infection site, MHC class II and CD11b positive neutrophils obtained from the footpad of infected BALB/c, LRP6^NKO^, LRP6^NKO^ DKK1^PKO^ and non-infected BALB/c mice were assessed. Compared to the infected BALB/c mice, we observed a significantly decreased MHC Class II and CD11b positive neutrophils in LRP6^NKO^ and LRP6^NKO^ DKK1^PKO^ infected mice (**Figures 2A, B**). Relative to the CD11b and MHC II negative neutrophils, CD11b and MHC II expression (MFI) significantly increased in CD11b and MHC II positive neutrophils (**Supplementary Figure S3**). Since less neutrophilic MHC class II and CD11b were found in LRP6^NKO^ and LRP6^NKO^ DKK1^PKO^ infected mice than in infected BALB/c mice, these data suggest that migration of activated neutrophils to the infection site is also dependent on the DKK1-LRP6 signalling pathway.

### Reduced myeloperoxidase-positive neutrophils in LRP6^NKO^ and LRP6^NKO^ DKK1^PKO^ infected mice

**Figure 3:**
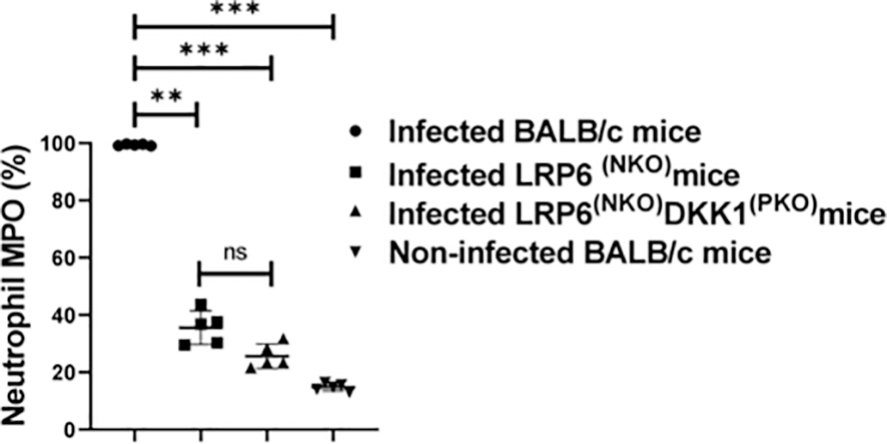
Decreased myeloperoxidase positive neutrophils derived from LRP6^NKO^ DKK1^PKO^ and LRP6^NKO^ infected mice on day 3 PI. The WT-BALB/c, LRP6^NKO^ and LRP6^NKO^ DKK1^PKO^ mice were challenged with infective metacyclic promastigote (2 x 10^6^ parasites, n = 5) of *L. major* via the footpad. Non-infected BALB/c mice (n = 10/2 feet per mouse) were given 0.9% NaCl saline. Neutrophils were isolated from the footpads of all infected and non-infected mice at day 3 PI. Isolated neutrophil samples were analyzed by flow cytometry for myeloperoxidase + cells. Each dot indicates myeloperoxidase + neutrophils, and the percentage of myeloperoxidase+ cells in the different experimental groups is shown in column graphs. Representative flow cytometry dot plots showing the analyses of myeloperoxidase + neutrophils and IgG1 isotype control for non-specific antibody staining, as well as a dot plot of each sample in all the experimental groups are presented in **Supplementary Figure S4**. In all the experiments, WT-infected and non-infected mice served as positive and negative controls, respectively. Results are presented as mean (± SEM). One-way ANOVA with Bonferroni’s *post hoc* test was performed to analyze the data **p < 0.01; ***p < 0.001; ns, non-significant (p > 0.05).

Myeloperoxidase (MPO) expression has also been proposed to mirror the degree of neutrophil activation (63–65). To further determine the possibility of DKK1 promoting recruitment of activated neutrophils to the infection site, the MFI of Ly6G+ neutrophils and MPO-positive neutrophils were determined from cells obtained from the footpad of infected WT BALB/c, LRP6^NKO^, LRP6^NKO^ DKK1^PKO^ and non-infected BALB/c mice. The MFI of Ly6G + cells and the number of neutrophils vary in the groups analyzed. A significantly higher number of neutrophils and increased neutrophilic expression of Ly6G was obtained from infected BALB/c mice compared to LRP6^(NPO)^, DKK1^(PKO)^, and LRP6^(NPO)^ infected mice (**Supplementary Figure S4**). Relative to the infected WT BALB/c mice, MPO-positive neutrophils were significantly reduced in LRP6^NKO^ and LRP6^NKO^ DKK1PKO-infected mice (**Figure 3**). Given the percentage of MPO-positive neutrophils in LRP6^NKO^ and LRP6^NKO^ DKK1^PKO^ infected mice is decreased in comparison to BALB/c infected mice, these data further confirm that the interaction of DKK1 and LRP6 is essential for the migration of activated neutrophils to the infection site. Interestingly, the levels of MPO, CD11b and MHC class II positive PMNs of LRP6^NKO^ and LRP6^NKO^ DKK1^PKO^ mice were comparable and were not significantly different from naïve mice, suggesting that the activation of PMNs upon infection is primarily through the LRP6 pathway.

### DKK1 signaling via LRP6 prolongs neutrophil longevity in infected BALB/c mice

**Figure 4:**
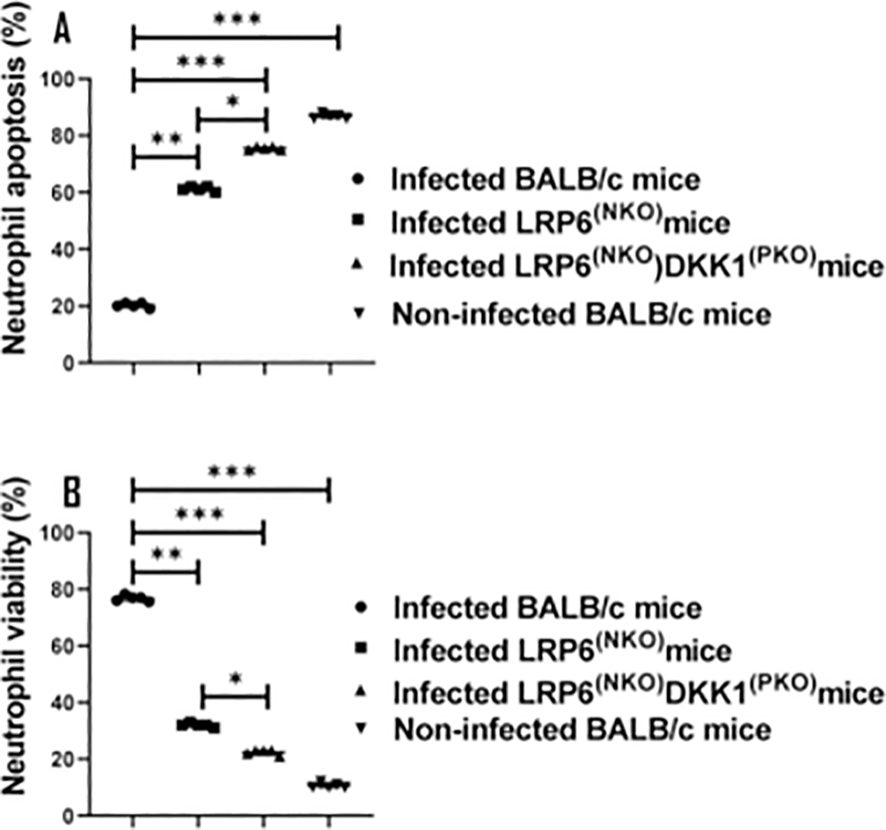
Infected LRP6^NKO^ and LRP6^NKO^ DKK1^PKO^ mice show elevated neutrophil apoptosis. The WT-BALB/c, LRP6^NKO^and LRP6^NKO^ DKK1^PKO^ mice were challenged with infective metacyclic promastigote (2 x 10^6^ parasites, n = 5) of *L. major* via the footpad. Non-infected BALB/c mice (n = 10/2 feet per mouse) were given 0.9% NaCl saline. Neutrophils were isolated from the footpads of all infected and non-infected mice at day 3 PI. Apoptotic neutrophils were analyzed by flow cytometry and identified by the increase in fluorescence intensity of annexin V-FITC. Each dot indicates either viable or apoptotic Ly6G+ cells. Percentage of apoptotic cells (early apoptosis: Annexin^+^, late apoptosis: Annexin^+^ PI^+^) in the infected and non-infected mice are shown in column graph **(A)** & neutrophil viability is shown in column graph **(B)**. Representative flow cytometry dot plots showing apoptotic and viable neutrophils after double staining with Annexin V-FITC and propidium iodide performed on day 3 PI. The first quadrant (Q1) represents necrotic neutrophils, the second quadrant (Q2) represents later apoptotic neutrophils, the third quadrant (Q3) represents early apoptotic neutrophils, and the fourth quadrant (Q4) represents normal neutrophils **Supplementary Figure S5**. A concatenated dot plot of each sample in all the experimental groups is presented in **Supplementary Figure S5**. In all the experiments, WT-infected and non-infected mice served as positive and negative controls, respectively. Results are presented as mean (± SEM). One-way ANOVA with Bonferroni’s *post hoc* test was performed to analyze the data *p < 0.05; **p < 0.01, ***p < 0.001.

*Leishmania* has the capacity to invade a variety of cell types- including neutrophils (66, 67). As a strategy to evade the immune response, as well as to achieve intracellular survival, it has been demonstrated that *Leishmania* inhibits cell apoptosis (16, 68). Given that DKK1 facilitates the infiltration of activated neutrophils to the infection site, we considered the possibility that platelet DKK1 induced by *L. major* infection blocks the apoptosis of the activated neutrophils. Thus, we challenged BALB/c, LRP6^NKO^, and LRP6^NKO^ DKK1^PKO^ mice with *L. major* and neutrophil apoptosis/viability was evaluated. Compared to infected BALB/c mice, the percentage of viable neutrophils (Q4) significantly decreased in infected LRP6^NKO^ and LRP6^NKO^ DKK1^PKO^ mice. The total apoptotic neutrophils (Q3 and Q2) in infected LRP6^NKO^ and LRP6^NKO^ DKK1^PKO^ mice was elevated. In contrast, there was a dramatic decrease in total neutrophil apoptosis in infected BALB/c mice (**Figures 4A, B**). These findings suggest that increased neutrophilic apoptosis at the infection site of LRP6^NKO^and LRP6^NKO^ DKK1^PKO^ mice may be mediated by the lack of DKK1 signaling via LRP6.

**Figure 5:**
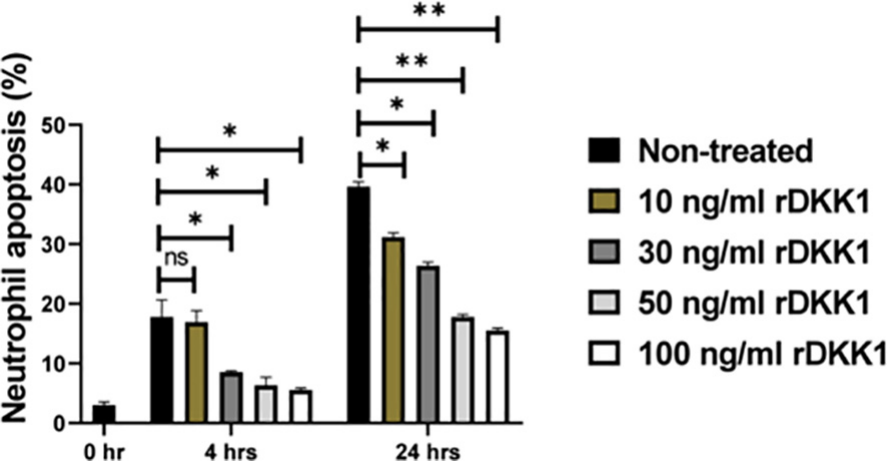
Recombinant DKK1 delayed neutrophil apoptosis in a dose-dependent manner. Neutrophils isolated from naïve mice were incubated with various concentrations of rDKK1. Neutrophil samples harvested at 0-, 4-, and 24 hrs post incubation were used to determine neutrophil apoptosis by flow cytometry. The percentage of apoptotic cells in the different experimental conditions is shown in the bar graph. Representative flow cytometry contour plots generated 24 hrs post incubation showed total apoptotic neutrophils (Q3 & Q2) after double staining with Annexin V-FITC and propidium iodide, as presented in **Supplementary Figure S6**. Also, a contour plot of each sample in all the experimental conditions is presented in **Supplementary Figure S6**. In all the experiments, non-treated neutrophils served as controls. Results are presented as mean (± SEM). One-way ANOVA with Bonferroni’s *post hoc* test was performed to analyze the data (*p < 0.05, **p < 0.01). ns, non-significant.

DKK1-induced delayed neutrophil apoptosis was further confirmed via *in vitro* stimulation of naïve neutrophils with rDKK1. Compared to non-treated cells, rDKK1 dose-dependently delayed neutrophil apoptosis (Q3 and Q2) between 4- and 24 hours post-treatment (**Figure 5**). Since the longevity of neutrophils is prolonged in the presence of rDKK1, these data provide direct evidence that DKK1 promotes neutrophil viability.

### Elevated expression of LRP6 in infected BALB/c mice

**Figure 6:**
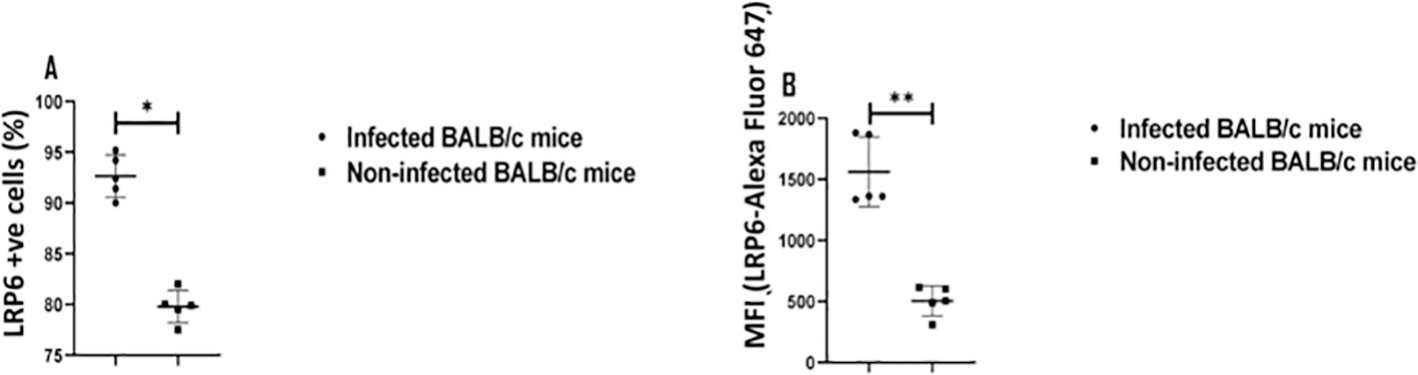
Elevated LRP6 expression in infected BALB/c mice. The WT-BALB/c mice were challenged with infective metacyclic promastigote (2 x 10^6^ parasites, n = 5) of *L. major* via the footpad. Non-infected BALB/c mice (n = 10/2 feet per mouse) were given 0.9% NaCl saline. Neutrophils were isolated from the footpads of infected and non-infected mice at day 3 PI. Isolated neutrophil samples were analyzed by flow cytometry for LRP6 expression. The column graph indicates the percentage of LRP6 positive cells **(A)** and the expression of LRP6 by Ly6G+ cells in the different experimental groups **(B)**. Representative flow cytometry dot plots showing the analyses of LRP6+ neutrophils and dot plots of each sample in all the experimental groups are presented in **Supplementary Figure S1**. Results are presented as mean (± SEM). Student’s t-test was performed to analyze the data (*p < 0.05; ** p < 0.01).

DKK1 has been established to interact with LRP5 and LRP6 (37, 69). Receptor density can be important in induction of signaling and cell activation. Although receptor recycling can lead to lowered levels of surface receptor expression as a result of ligand activation, we examined the level of LRP6 expression on PMNs during infection. To determine the neutrophil expression of LRP6 during infection, cells were harvested from non-infected and infected BALB/c mice. Infected LRP6^NKO^ and LRP6^NKO^ DKK1^PKO^ mice were used as negative expression controls (data not shown). Relative to non-infected BALB/c mice, the percentage of neutrophils positive for LRP6 and LRP6 mean fluorescent intensity in infected BALB/c mice are significantly increased (**Figures 6A, B**). These findings support that DKK1 signaling occurs through the canonical LRP6 receptor, which is further upregulated in murine PMNs in response to *Leishmania* infection. The mechanism involved in the upregulation of LRP6 expression is unclear but may be the result of cytokines or other mediators produced in response to infection as well as DKK1 itself (70). However, LRP6 upregulation would serve to increase the role of this pathway.

### Reduction of parasitic load in infected LRP6^NKO^ and LRP6^NKO^ DKK1^PKO^ mice on day 14 PI

**Figure 7:**
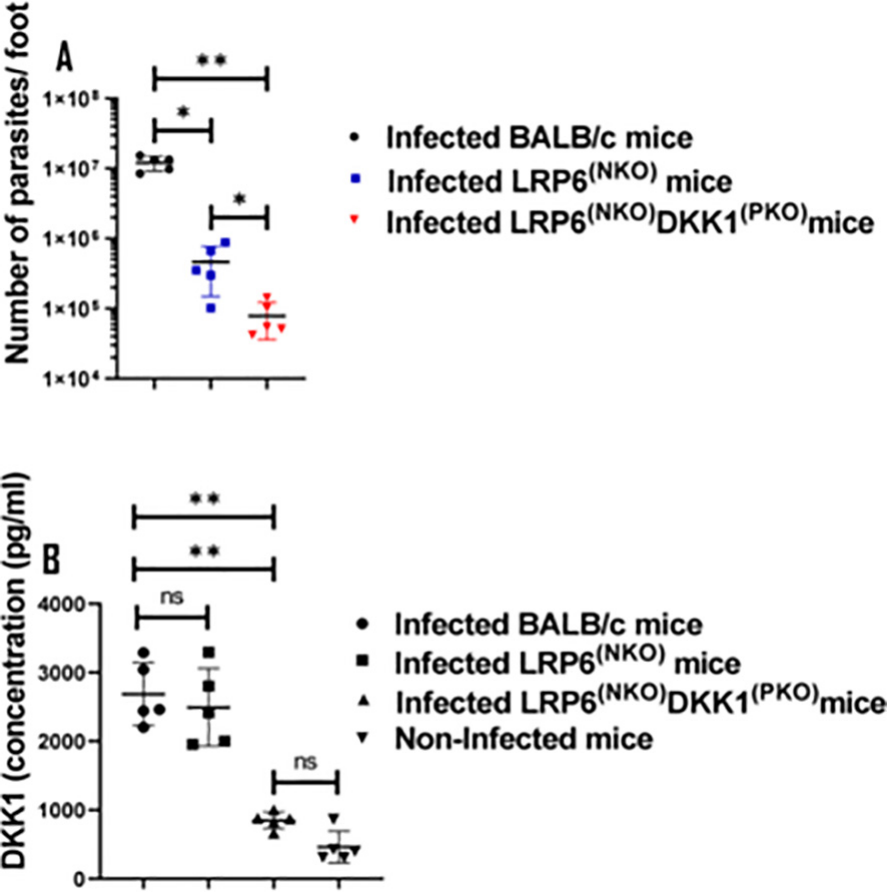
Parasite load in the infected LRP6^NKO^ and LRP6^NKO^ DKK1^PKO^ mice were significantly reduced, while plasma DKK1 production in infected BALB/c and LRP6^NKO^ mice were comparable. BALB/c, LRP6^NKO^ and LRP6^NKO^ DKK1^PKO^ mice were challenged with infective metacyclic promastigote (2 x 10^6^ parasites, n = 5) of WT *L. major* strain via the footpad. Control mice (n = 10/2 feet per mouse) were given 0.9% NaCl Saline. The infected foot from each mouse was used to determine parasite load using a limiting dilution assay on day 14 PI. Parasite load in the infected BALB/c group was compared with the infected LRP6^NKO^ and LRP6^NKO^ DKK1^PKO^ mice. To determine DKK1 production, blood was collected via the maxillary vein at day 3 PI. Plasma samples were analyzed by ELISA. In all experiments, Infected and non-infected BALB/c mice served as positive and negative controls, respectively. Results of parasite load are presented as mean +/- SEM, and data analysis was done using one-way ANOVA with Bonferroni’s *post hoc* test *p < 0.05, **p < 0.01, (p > 0.05). For DKK1 concentration, results are presented as mean +/- SEM of replicate wells. One-way ANOVA with Bonferroni’s *post hoc* test was performed to analyze the data **p < 0.01, ‘ns’ indicates not significant (p > 0.05).

Previous studies reported that the internalization of viable *Leishmania* parasites is a potent inducer of delayed neutrophil apoptosis (16). Thus, we speculated that the decreased percentage of apoptotic neutrophils in BALB/c-infected mice may be associated with increased parasitic load. As anticipated, the parasitic load was significantly decreased in LRP6^NKO^and LRP6^NKO^ DKK1^PKO^ infected mice compared to infected BALB/c mice (**Figure 7A**). These findings indicate that *Leishmania* infection delayed spontaneous neutrophil apoptosis by inducing DKK1 production in infected BALB/c mice; further activation by DKK1 and a direct inhibition of PMN apoptosis also can contribute to enhanced parasite survival.

We previously showed that LPG is a virulence factor of *L. major* that induces DKK1 production from activated platelets through TLR2 (19). Furthermore, the high concentration of DKK1 produced in response to *L. major* infection was found to decrease following antibody-mediated platelet depletion (31). To confirm that the effect seen in infected BALB/c mice is solely dependent on platelet DKK1 signalling via LRP6, we examined the plasma production of DKK1 in WT and LRP6^NKO^ and LRP6^NKO^ DKK1^PKO^ mice. Results showed comparable production of DKK1 in infected LRP6^NKO^and BALB/c mice (**Figure 7B**). Since similar levels of DKK1 were observed in infected LRP6^NKO^and BALB/c mice, these data suggest that infection as well as the sustenance of activated neutrophils in infected BALB/c mice is dependent on the PMN DKK1-LRP6 signalling. Interestingly, LRP6^NKO^ DKK1^PKO^ mice have a slightly lower parasite burden than mice whose PMNs are deficient in LRP6 alone, suggesting that other targets of DKK1 may contribute to disease. However, the primary effect appears to be through PMN-DKK1 activation.

## Discussion

Upon parasite inoculation via needle injection or sandfly bite, neutrophils are rapidly recruited to the inoculation site from the bloodstream, representing the first cells infected by *Leishmania* promastigotes (11). Experimentally, parasitized neutrophils were observed histologically within 6 hours following footpad injection of *L. major* in both BALB/c and C57Bl/6 mice (71). The different factors derived from the parasite are all involved in the infiltration of cells and the onset of infection. For instance, *Leishmania* promastigotes were shown to actively promote neutrophil recruitment via endothelial cell production of granulocyte chemotactic factor and CXCL8 (effective chemoattractant for neutrophil) (72, 73). Further, host factors can actively induce neutrophil recruitment. Indeed, neutrophils are highly responsive to members of the CXC chemokine family, such as IL-8, which is reported to contribute to early neutrophil recruitment at the site of parasite inoculation in humans (74, 75). Neutrophil recruitment can also occur via cytokines such as tumor necrosis factor (TNF) and interleukin 17 (IL-17) (76–79). It has been established that these cytokines are not the major contributor to very early neutrophil recruitment but appear to be involved in facilitating neutrophil influx into the infection site at a later phase of infection (75, 79). Thus, the primary immune mechanisms governing the intensified neutrophil recruitment at the infection site during the early phase of infection and disease development (days 3-14) is not completely understood. Previously, we demonstrated that LPG expressed by metacyclic promastigotes induces DKK1 production from platelets through the activation of TLR2 within 72 hours post-infection (19), and DKK1 released promoted leukocyte-platelet aggregation, which is essential for early trafficking of neutrophils and polarization of immune responses in pathological type 2 cell-mediated inflammation (19, 31). Since the expression of *Leishmania-*derived LPG wanes with differentiation into the amastigote by 72 hours, the underlying mechanism regulating cell migration to an inflammatory site beyond 72 hours post-infection remained undefined. To gain a more comprehensive and mechanistic understanding of this process, this study evaluates further the mechanisms through which parasitic and host factors mediate the infiltration and longevity of neutrophils at the infection site.

We initially focused on assessing the contribution of *Leishmania* PAMPs to the migration of neutrophils to the infection site by comparing *L. major* deficient in LPG synthesis (*Δlpg1^-^*) or lacked all ether phospholipids, including plasmalogens, LPG, and GIPL**s** (*Δads1^-^*). Interestingly, the deletion of LPG in the *Δlpg1^-^* mutant or the absence of all ether phospholipids, in the *Δads1^-^* mutant significantly impair the leukocyte platelet aggregation and migration of PMN to the infection site compared to the responses obtained from WT *L. major* infected mice. This implied that in *Δlpg1^-^* mutant infected mice, the significantly low percentage of neutrophils on day 3 PI is likely related to the absence of LPG, and the elevated neutrophils on 7- and 14-days PI are linked to the expression of other surface molecules associated with the amastigote stage. This is consistent with our previous studies that showed lack of LPA formation induced by *Δlpg1^-^* parasites is restored in addback parasite (*Δlpg1-*/+LPG1) infected mice at day 3 PI (19). Similarly, the low level of activated neutrophils in *Δads1^-^* parasite-infected mice beyond day 3 PI suggest that parasite ether phospholipids may be the primary factor influencing the trafficking of neutrophils to the infection site after promastigote transformation. Notably, GIPLs are expressed on all parasite stages and have been found to stimulate the host immune response (26, 80); however, the immunologic roles of various *Leishmania* ether phospholipids remain largely unexplored. Thus, these results provide evidence that multiple *Leishmania* PAMPs-induce local inflammatory signals which may contribute to the continual migration of activated neutrophils to the infection site.

In this study, we also demonstrated that neutrophils expressed LRP6, and the DKK1-LRP6 interaction promotes the infiltration of activated neutrophils in the infection site of BALB/c mice. This observation is in accordance with our previous studies, which showed that DKK1 upregulates LPA and leukocytes in blood obtained from wild-type parasite-infected mice, and pretreatment with a DKK1 inhibitor prior to infection with *L. major* reduced the elevation of LPA formation, as well as subsequent neutrophil infiltration to the draining lymph node (31). This finding suggests that DKK1-LRP6 signalling regulates migration and the full effector function of neutrophils. Although the LPA levels at the site of infection were not examined in that study, however, it is likely that the systemic response would also be observed at the site of infection.

To evade the host immune response and survive intracellularly, *Leishmania* extend the life span of neutrophils by inhibiting neutrophil spontaneous apoptosis (68). Although *Leishmania* inhibits apoptosis of various host cell types, delayed neutrophil apoptosis is arguably critical because it represents an early acute inflammatory response that regulates the outcome of infection. Because the DKK1-LRP6 signalling pathway promotes infiltration of activated neutrophils in the infection site, we tested the hypothesis that DKK1-LRP6 interaction can extend the life span of neutrophils. The increased viable neutrophils obtained from infected BALB/c mice suggest that DKK1-LRP6 signalling influences the longevity of neutrophils. This effect was further established *in vitro* using neutrophils treated with rDKK1. We did not specifically examine whether viable neutrophils were all infected. Other *in vivo* studies have however found that viable neutrophils are both infected and non-infected (81). *Leishmania* infection has been shown previously to delay cell apoptosis (16). Thus, this process can be multi-factorial. However, as shown in our study, the action of DKK1 can impact neutrophil apoptosis. Further, the LRP6 upregulation in infected BALB/c would serve to promote the role of the DKK1-LRP6 signalling pathway. However, the underlying process involved in LRP6 upregulation in infected BALB/c mice is unknown, and it may involve other host factors released during infection. The increased parasitic burden found in infected BALB/c mice suggests that elevated neutrophil activation and inhibition of PMN apoptosis are important in parasite survival and progression. This is consistent with previous studies which demonstrated the presence of internalized viable parasites as a potent inducer of delayed neutrophil apoptosis (16). However, another underlying mechanism for deterring PMN apoptosis appears to be receptor-mediated (LRP6). It is possible that the non-apoptotic neutrophils obtained from infected BALB/c mice include neutrophils that ingested *Leishmania* in the first hours post-infection and whose apoptosis was delayed initially by DKK1 released by the parasite-activated platelet. Interestingly, the comparable production of DKK1 in infected LRP6^NKO^and BALB/c mice further confirms that the neutrophil continual migration and longevity in infected BALB/c mice is primarily dependent on platelet DKK1 signalling via LRP6.

In conclusion, our study elucidates the role of DKK1 and parasitic components in regulating the infiltration and longevity of neutrophils at the infection site. Since we previously established that DKK1 is a potent inducer of type 2 cell-mediated immune responses (31), it is possible that the lack of LRP6-DKK1 signaling in neutrophils obtained from the mutant mice could reduce the type 2 immune response and have a protective effect. Thus, the role of LRP6-DKK1 would be of interest and further define the T-cell-neutrophil-platelet dynamic in *Leishmania* infection.

## Data Availability

The original contributions presented in the study are included in the article/**Supplementary Material**. Further inquiries can be directed to the corresponding author.
