# Supplementary material for: Leishmania major surface components and DKK1 signalling via LRP6 promote migration and longevity of neutrophils in the infection site
Source: Front Immunol. 2024 Oct 22;15:1473133. doi: 10.3389/fimmu.2024.1473133 (PMC11534728; doi:10.3389/fimmu.2024.1473133)
Supplement: Supplementary file 1 [file Table1.docx]

**Supplementary Material**

***Leishmania major-*surface components and DKK1 signalling via LRP6 promote migration and longevity of neutrophils in the infection site**

**Olivia C. Ihedioha^1^, Haley Marcarian^1^, Anutr Sivakoses^1^, Stephen M. Beverley^2^, Diane McMahon-Pratt^3^, Alfred L.M. Bothwell^1^**

*** Correspondence:** Alfred L.M. Bothwell: [albothwell@unmc.edu](mailto:albothwell@unmc.edu)


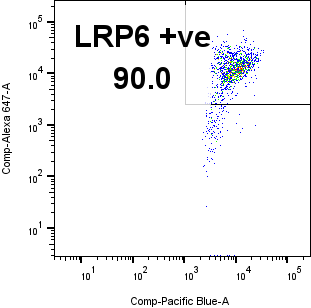

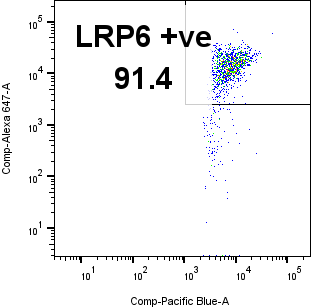

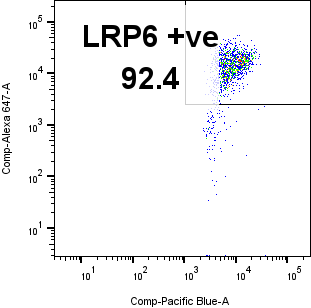

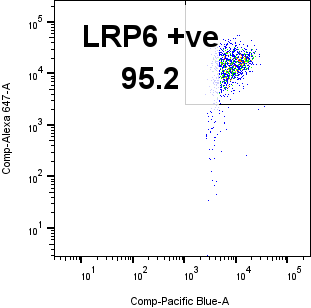

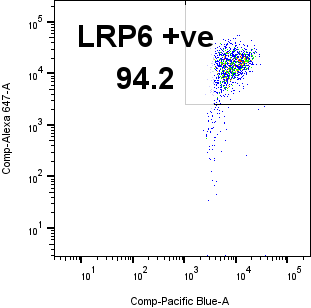

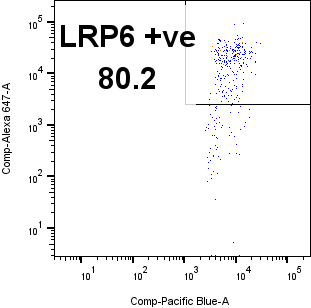

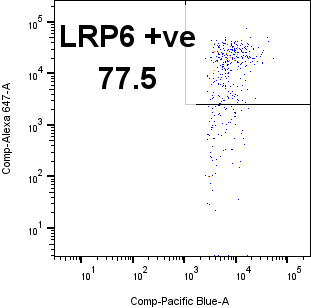

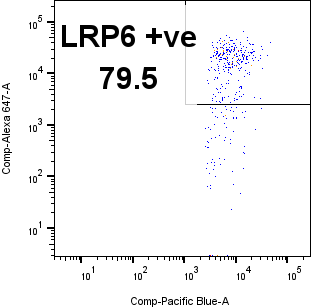

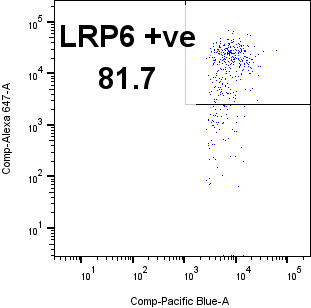

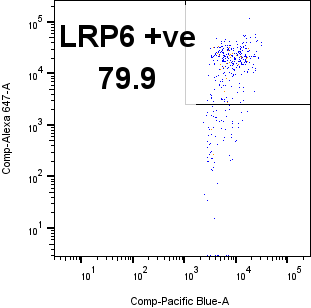


**LRP6-Alexa Fluor 647**

**Ly6G-Pacific Blue**

**Non-infected**

**BALB/c mice**

**Infected**

**BALB/c mice**


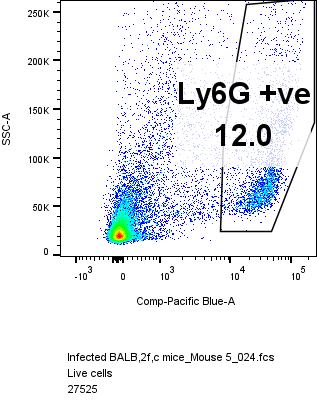

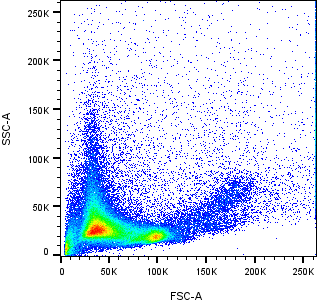

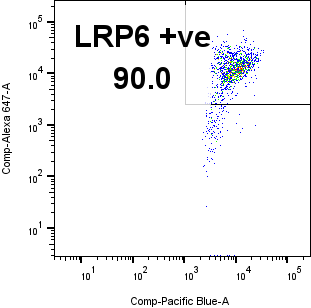


**SSC-A**

**FSC-A**

**Ly6G-Pacific Blue**

**SSC-A**

**Ly6G-Pacific Blue**

**LRP6-Alexa Fluor 647**

**A**

**A**

**B**

**Fig. S1: Elevated cell surface LRP6 in infected BALB/c mice.** Infected BALB/c mice (5/group) were challenged with infective metacyclic promastigote (2 x 10^6^ parasites, n = 5) of WT strain via the footpad. Control mice (n = 10/2 feet per mouse) were given 0.9% NaCl saline. Cells from the infected footpad were collected on day 3 PI. Representative flow cytometry dot plots showing the analyses of LRP6+ neutrophils **(A),** and the percentage of LRP6+ cells in all the experimental groups are presented in **(B).**


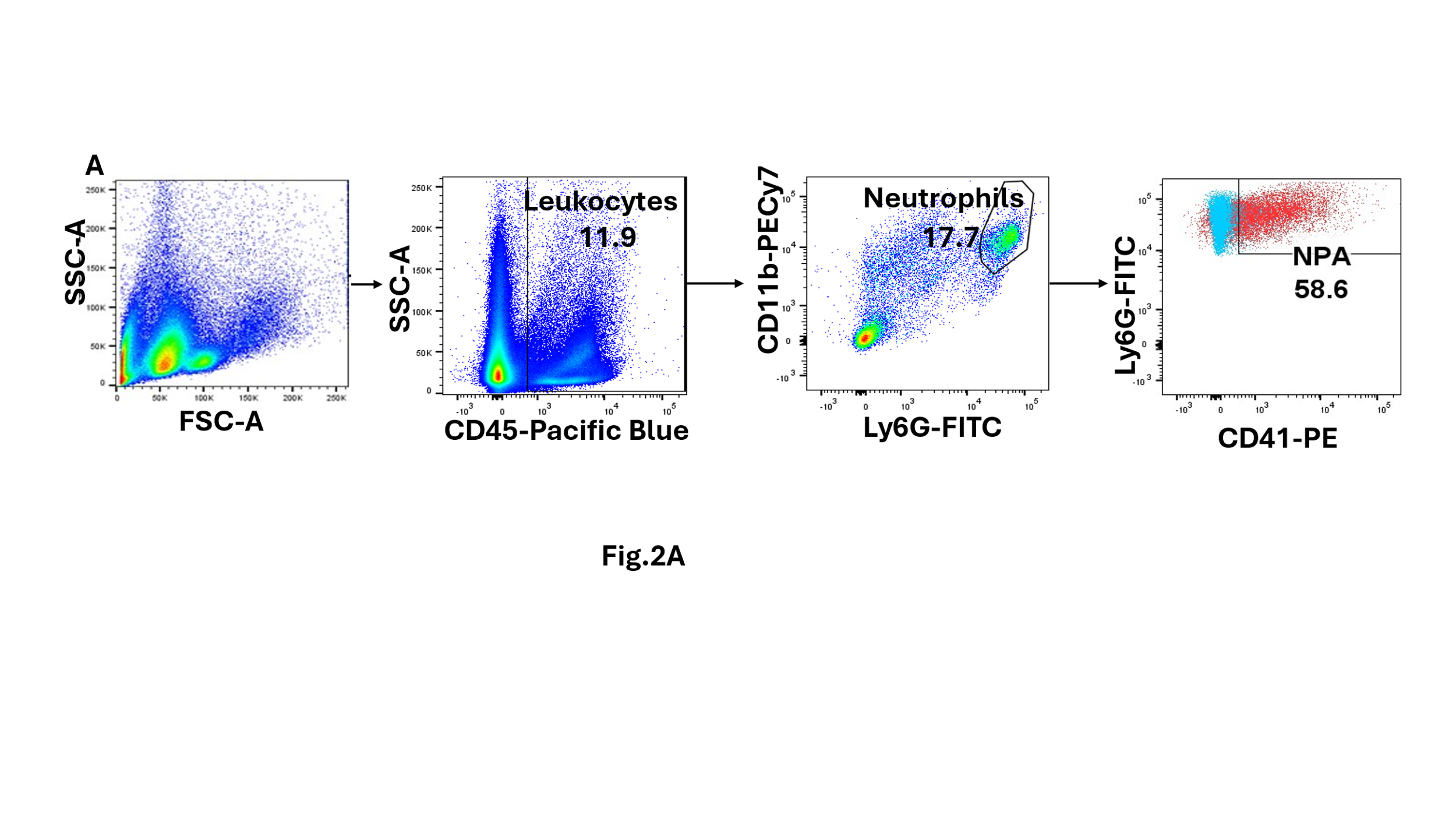


**B**


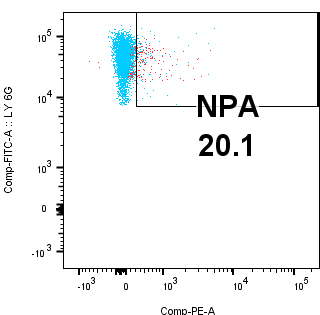

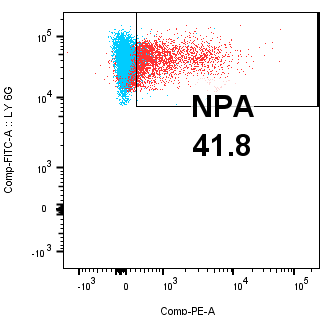

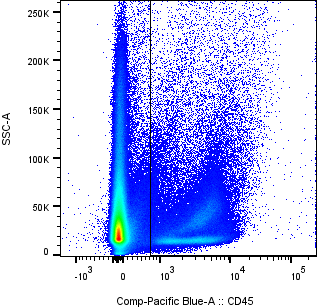


**Leukocytes**

**9.1**


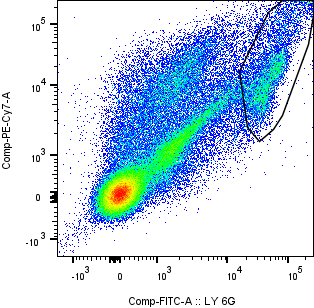


**Neutrophils**

**6.9**


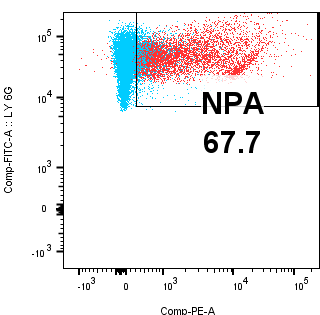

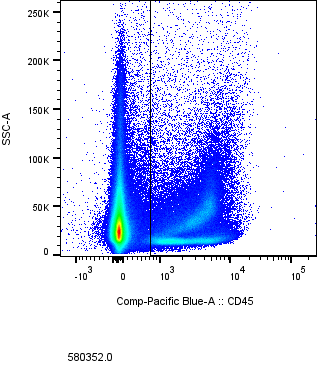


**Leukocytes**

**14.4**


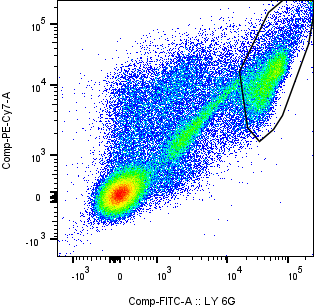


**Neutrophils**

**17.9**

**WT-infected**

***Δads1^-^* infected**

**CD41**


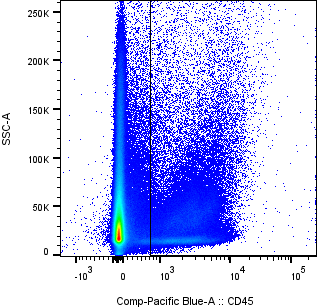


**Leukocytes**

**6.0**


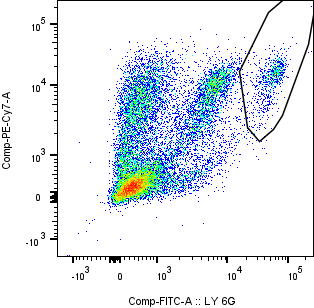


**Neutrophils**

**3.7**

**SSC-A**

**Ly6G**

**CD11b**

**CD45**

**Ly6G**

**Non-infected**

**Day 3 PI**

**A**

**C**


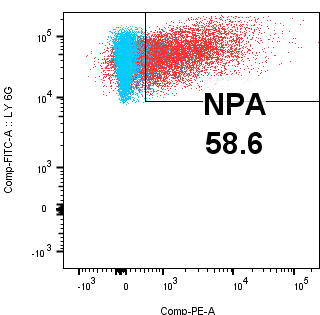

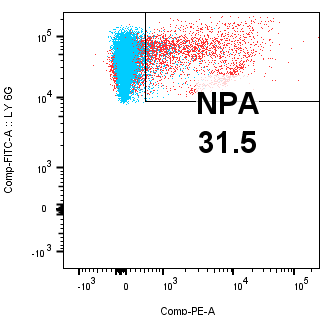

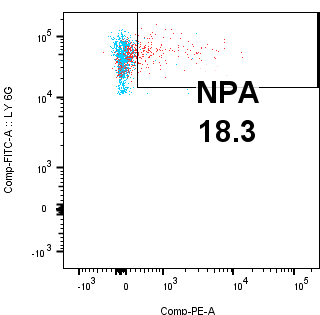

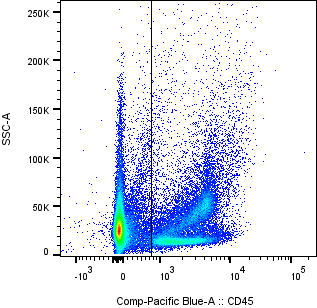


**Leukocytes**

**23.2**


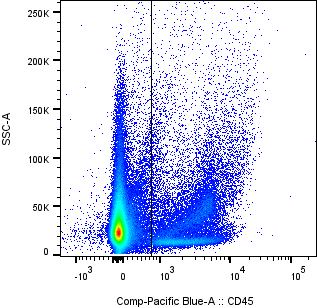


**Leukocytes**

**10.7**


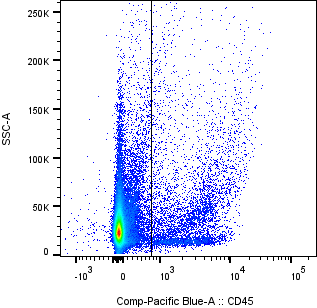


**Leukocytes**

**2.7**


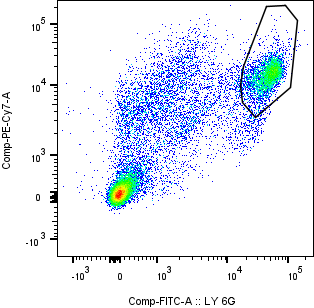


**Neutrophils**

**17.7**


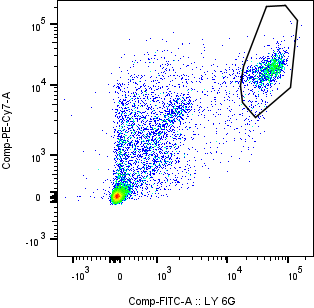


**Neutrophils**

**10.1**


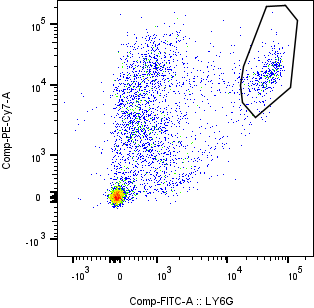


**Neutrophils**

**7.3**

**SSC-A**

**Ly6G**

**CD11b**

**CD45**

**Ly6G**

**CD41**

**Day 7 PI**

**WT-infected**

***Δads1^-^* infected**

**Non-infected**

**D**


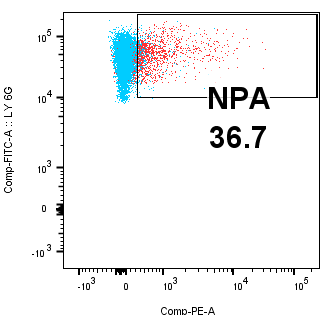

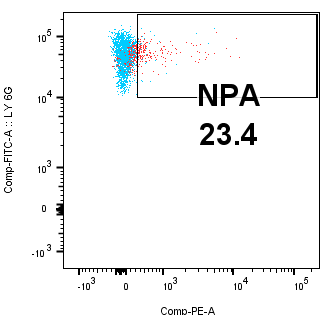

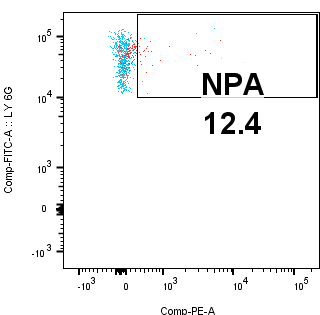

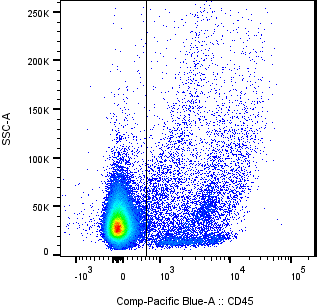


**Leukocytes**

**5.6**


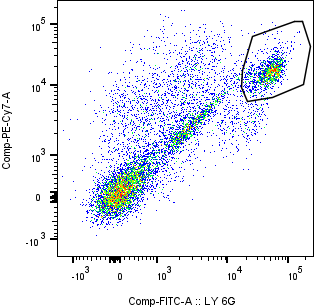


**Neutrophils**

**12.4**

**SSC-A**

**Ly6G**

**CD11b**

**CD45**

**Ly6G**

**CD41**


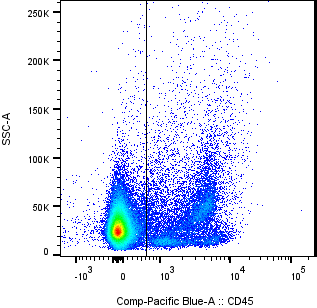


**Leukocytes**

**12.9**


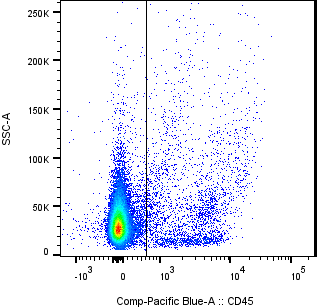


**Leukocytes**

**4.1**


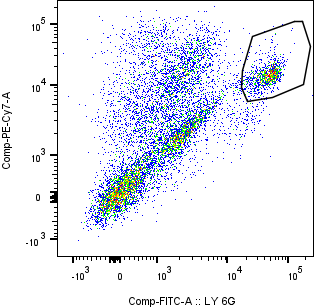


**Neutrophils**

**7.5**


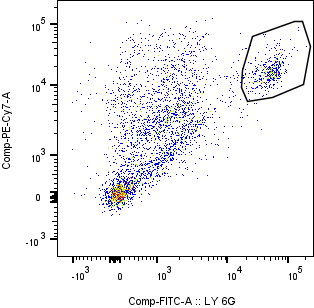


**Neutrophils**

**4.9**

**Day 14 PI**

**WT-infected**

***Δads1^-^* infected**

**Non-infected**

**E**


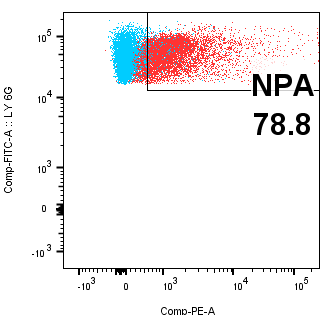

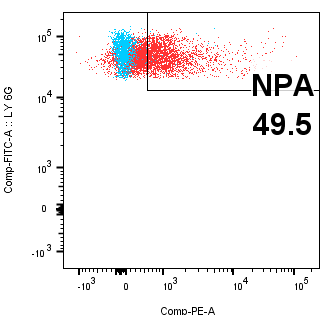

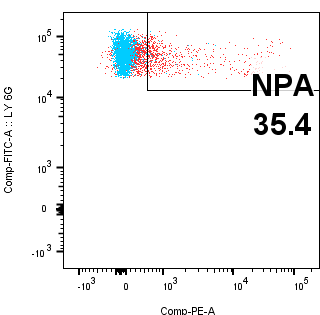

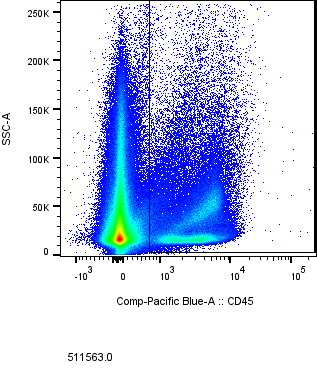


**Leukocytes**

**16.2**


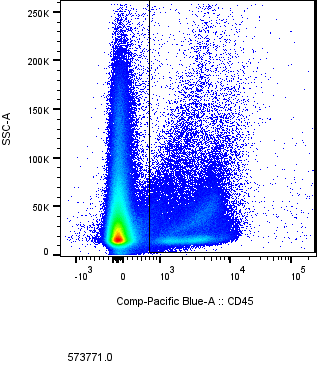


**Leukocytes**

**9.9**


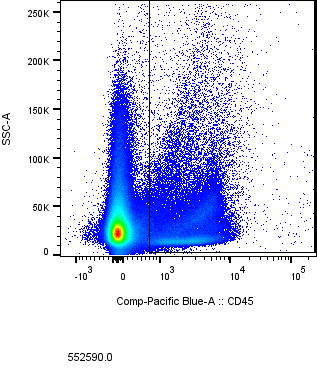


**Leukocytes**

**8.7**


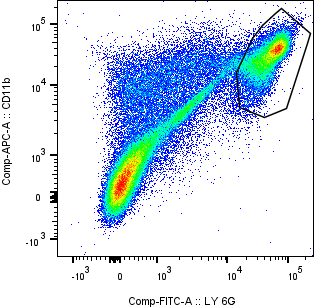


**Neutrophils**

**22.5**


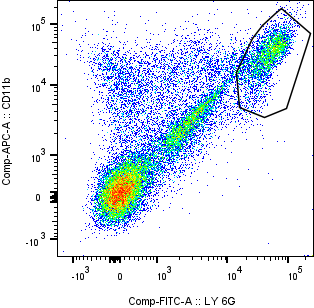


**Neutrophils**

**12.4**


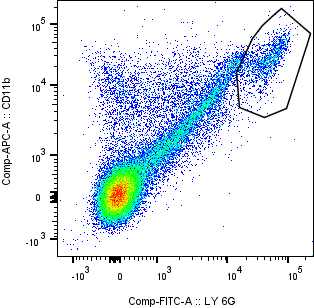


**Neutrophils**

**5.8**

**Non-infected**

**WT-infected**

***Δlpg1^-^* infected**

**SSC-A**

**CD11b**

**Ly6G**

**Ly6G**

**CD45**

**CD41**

**Day 3 PI**

**F**


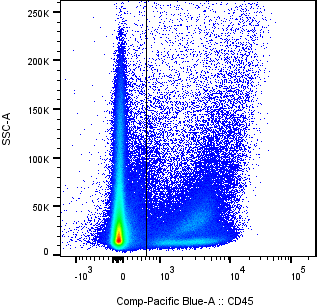

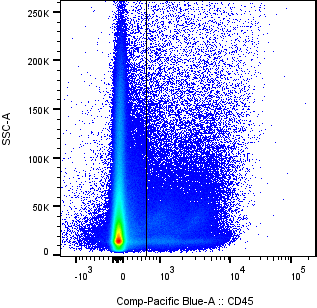

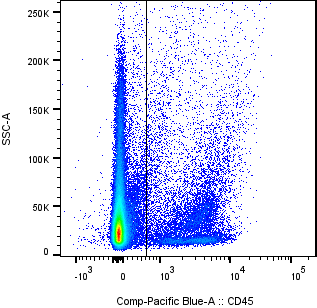

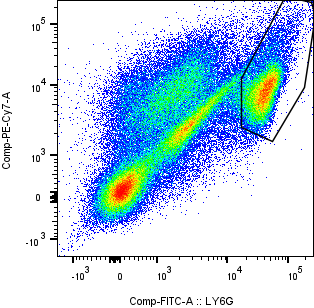

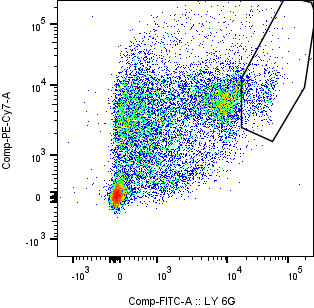

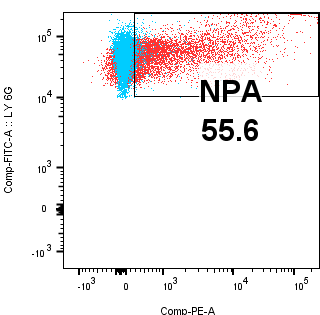

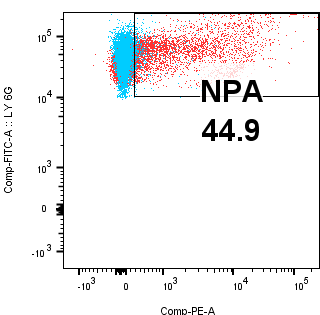

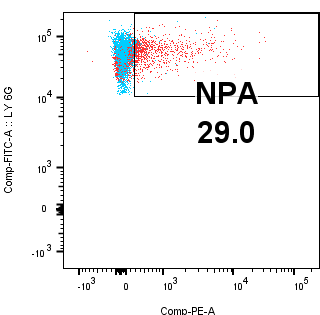


**Leukocytes**

**10.4**

**Leukocytes**

**7.1**

**Leukocytes**

**5.4**

**Neutrophils**

**22.0**

**Neutrophils**

**8.0**

**Non-infected**

**WT-infected**

***Δlpg1^-^* infected**

**Ly6G**

**CD45**

**CD41**

**SSC-A**

**CD11b**

**Ly6G**

**Day 7 PI**


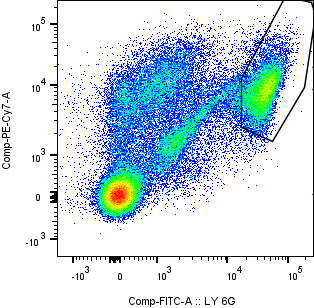


**Neutrophils**

**22.0**

**G**


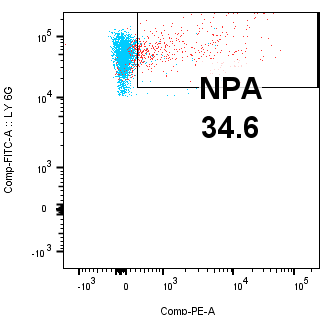

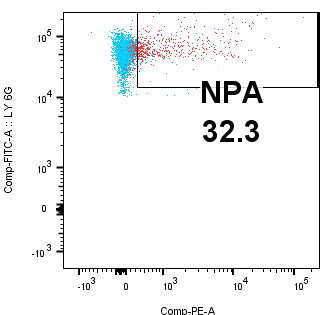

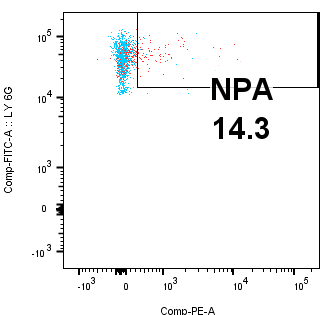

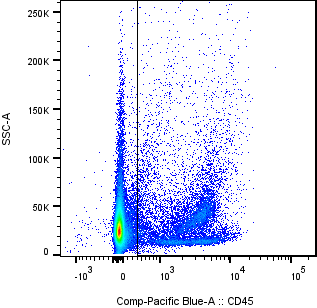


**Leukocytes**

**10.6**


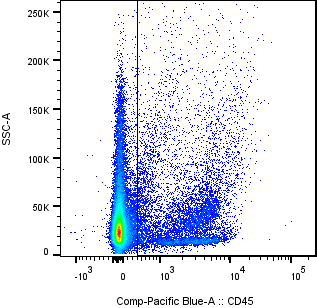


**Leukocytes**

**7.7**


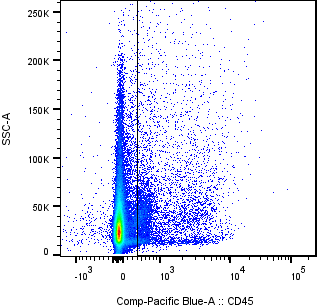


**Leukocytes**

**3.0**


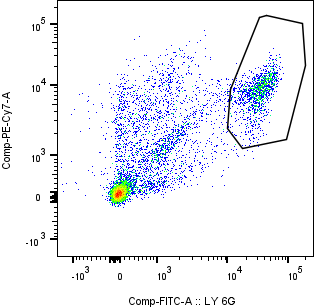


**Neutrophils**

**18.3**


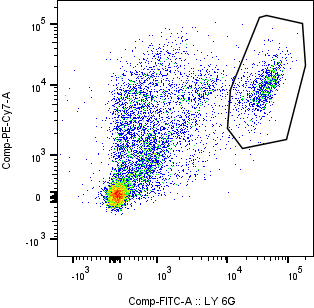


**Neutrophils**

**15.4**


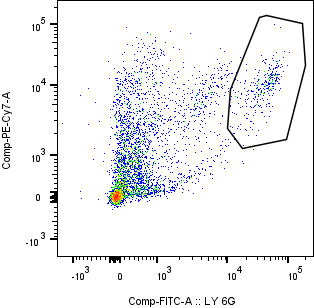


**Neutrophils**

**7.5**

**Non-infected**

**WT-infected**

***Δlpg1^-^* infected**

**Ly6G**

**CD45**

**CD41**

**SSC-A**

**CD11b**

**Ly6G**

**Day 14 PI**

**Fig. S2: *Δlpg1*- and *Δads1^-^* parasites are less effective in inducing neutrophil infiltration and neutrophil platelet aggregates (NPA)**

BALB/c mice (5/group) were challenged with infective metacyclic promastigote (2 x 10^6^ parasites, n = 5) of WT, *Δlpg1*- and *Δads1^-^* strains via the footpad. Control mice (n = 10/2 feet per mouse) were given 0.9% NaCl saline. Cells from the infected footpad were collected on days 3, 7 and 14 PI. Samples were analyzed by flow cytometry for neutrophils and NPA formation. Representative flow cytometry dot plots showing the analyses of neutrophils and NPA **(A)**, the percentage of neutrophils and NPA molecules obtained from concatenated samples of each experimental group are indicated **(B)**, **(C)**, **(D)**, **(E)**, **(F)**, & **(G)**. In all the experiments, WT-infected and non-infected mice served as positive and negative control, respectively.

**A**


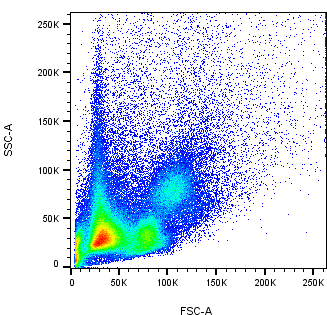


**FSC-A**

**SSC-A**

**SSC-A**

**Ly6G-FITC**

**Ly6G-FITC**

**Ly6G-FITC**

**CD11b-Alexa Fluor 700**

**MHC II-PE**


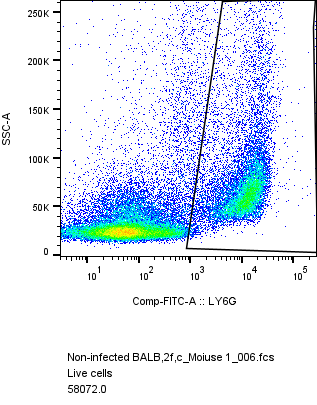


**Ly6G +ve**

**24.1**


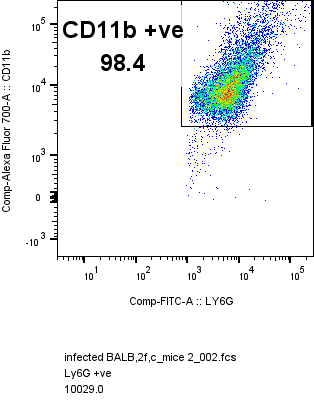

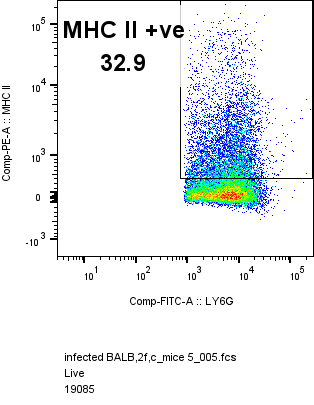


**B**


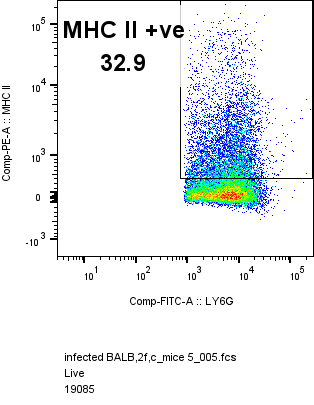

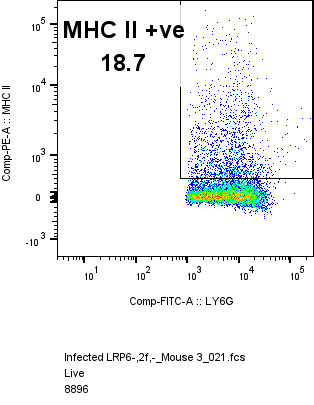

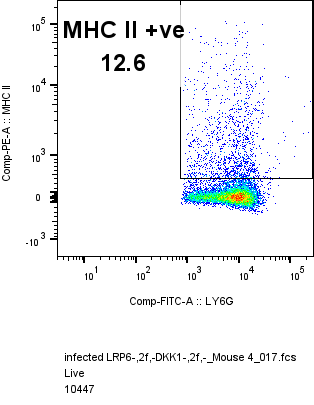

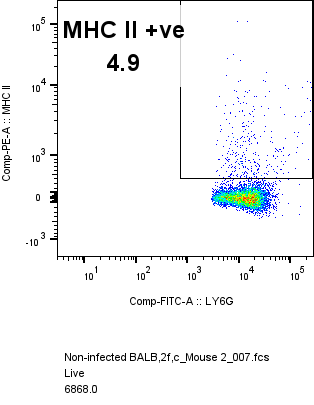

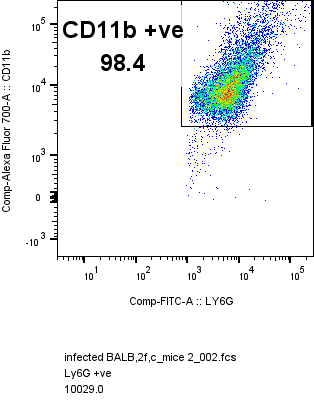

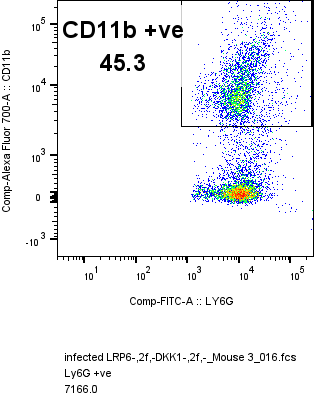

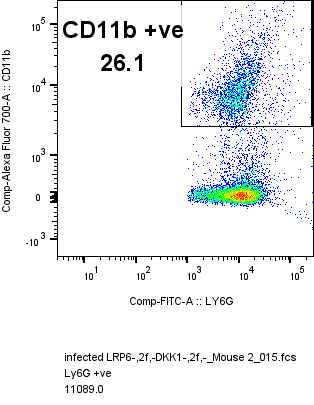

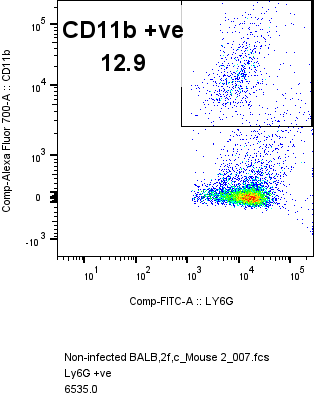


**CD11b**

**Ly6G**

**MHC class II**

**SSC-A**

**Infected LRP6^(NKO)^**

**mice**

**Non-infected**

**mice**

**Infected LRP6^(NKO)^**

**DKK1^(PKO)^mice**

**Infected BALB/c**

**mice**


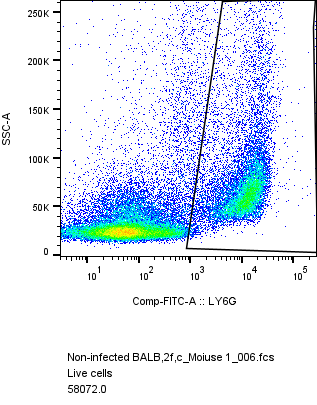


**Ly6G +ve**

**24.2**


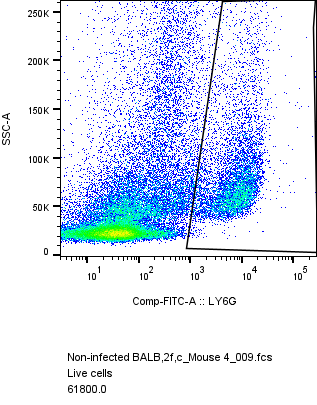


**Ly6G +ve**

**14.6**


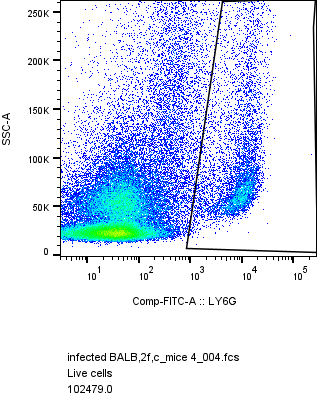


**Ly6G +ve**

**9.4**


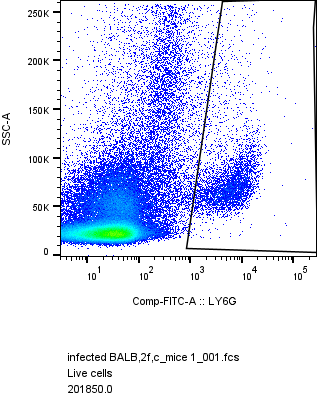


**Ly6G +ve**

**2.5**

**C**

**D**

**Fig. S3: Impaired CD11b and MHC positive neutrophils obtained from** **LRP6^(NPO)^ DKK1^(PKO)^ and LRP6^(NPO)^ infected mice**

Infected BALB/c, LRP6^NKO^ DKK1^PKO^ and LRP6^NKO^ mice were challenged with infective metacyclic promastigote (2 x 10^6^ parasites, n = 5) of WT strain via the footpad. Control mice (n = 10/2 feet per mouse) were given 0.9% NaCl saline. Cells from the infected footpad were collected on day 3 PI. Samples were analyzed by flow cytometry for CD11b and MHC II positive cells. Representative flow cytometry dot plots showing the analyses of CD11b and MHC class II + neutrophils performed on day 3 PI **(A)**. The percentage of CD11b and MHC class II + neutrophils obtained from concatenated samples of each experimental group are indicated **(B)**. The mean fluorescent intensity (MFI) of CD11b and MHC II expressed by CD11b and MHC II positive /negative neutrophils is presented in column graphs **(C)** & **(D)**. Ly6G +ve cells indicate neutrophils. In all the experiments, infected and non-infected BALB/c mice served as positive and negative controls, respectively.


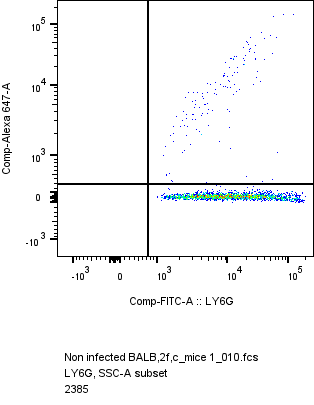


**Q2**

**4.5**

**Q3**

**95.5**

**Q1**

**0.0**

**Q4**

**0.0**


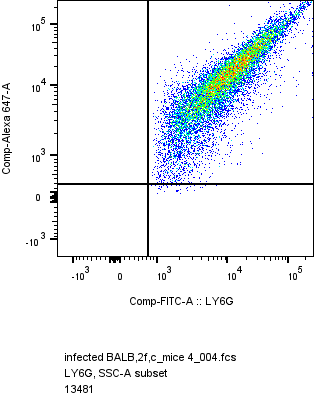


**Q1**

**0.1**

**Q2**

**99.1**

**Q4**

**0.0**

**Q3**

**0.7**


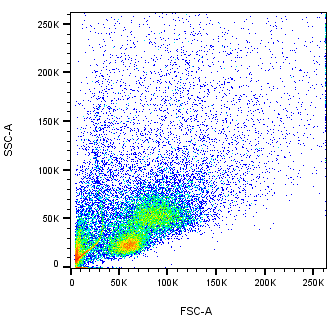


**FSC-A**

**SSC-A**

**Ly6G-FITC**

**Ly6G-FITC**

**IgG1-Alexa Fluor 647**

**MPO-Alexa Fluor 647**

**A**

**Ly6G-FITC**


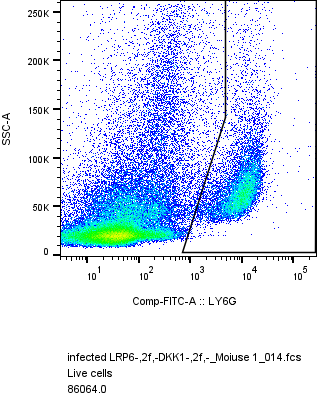


**Ly6G +ve**

**15.2**


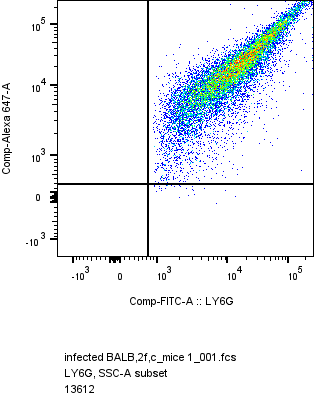

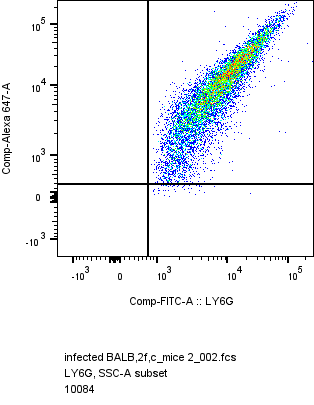

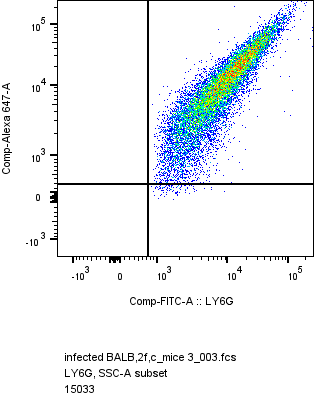

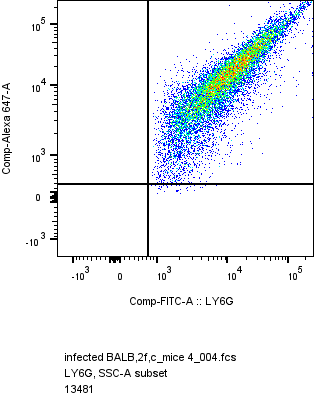

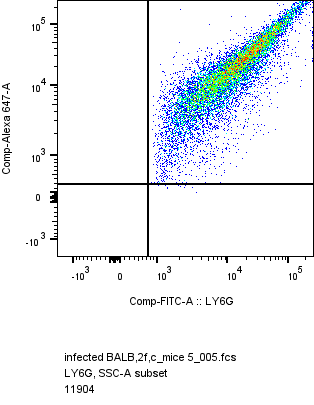

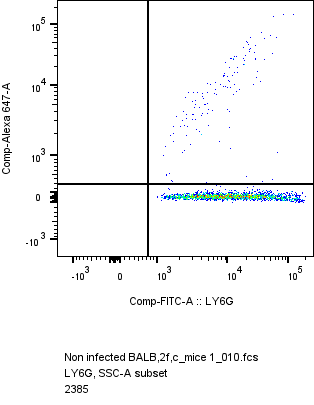

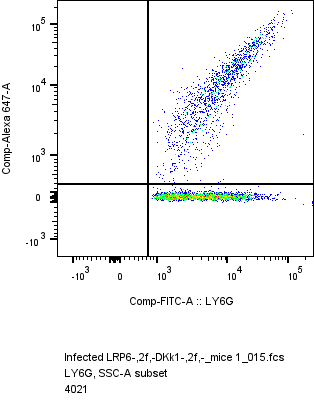

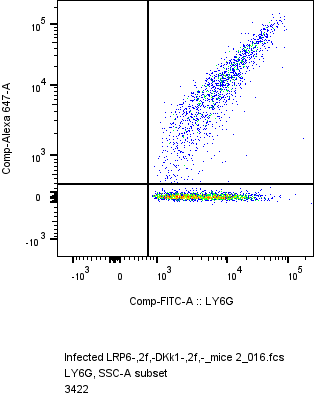

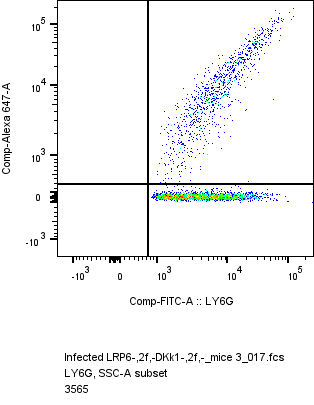

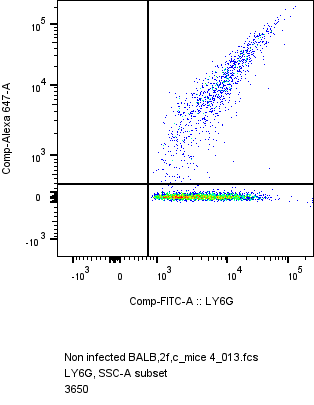

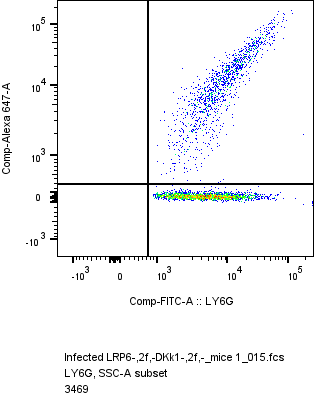

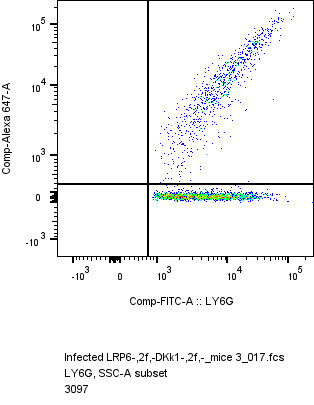


**MPO-Alexa Fluor 647**

**Ly6G-FITC**

**IgG1 Alexa Fluor 647**

**Ly6G-FITC**

**Infected**

**BALB/c mice**

**Infected**

**LRP6^(NPO)^ mice**

**Non-infected**

**BALB/c mice**

**IgG1 Isotype control**

**Q1**

**0.2**

**Q2**

**99.6**

**Q3**

**0.2**

**Q4**

**0.0**

**Q1**

**0.1**

**Q2**

**99.1**

**Q4**

**0.0**

**Q3**

**0.7**

**Q1**

**0.2**

**Q2**

**99.4**

**Q4**

**0.0**

**Q3**

**0.4**

**Q1**

**0.1**

**Q1**

**0.2**

**Q4**

**0.0**

**Q4**

**0.0**

**Q2**

**99.7**

**Q2**

**99.0**

**Q3**

**0.1**

**Q3**

**0.8**

**Q1**

**0.0**

**Q1**

**0.0**

**Q1**

**0.0**

**Q1**

**0.0**

**Q1**

**0.0**

**Q4**

**0.0**

**Q4**

**0.0**

**Q4**

**0.0**

**Q4**

**0.0**

**Q4**

**0.0**

**Q2**

**36.8**

**Q2**

**29.5**

**Q2**

**47.3**

**Q2**

**30.3**

**Q2**

**37.6**

**Q3**

**63.2**

**Q3**

**70.5**

**Q3**

**69.7**

**Q3**

**56.3**

**Q3**

**62.4**

**Q1**

**0.0**

**Q1**

**0.0**

**Q1**

**0.0**

**Q1**

**0.0**

**Q1**

**0.0**

**Q1**

**0.0**

**Q1**

**0.0**

**Q1**

**0.0**

**Q1**

**0.0**

**Q1**

**0.0**

**Q4**

**0.0**

**Q4**

**0.0**

**Q4**

**0.0**

**Q4**

**0.0**

**Q4**

**0.0**

**Q4**

**0.0**

**Q4**

**0.0**

**Q4**

**0.0**

**Q4**

**0.0**

**Q4**

**0.0**

**Q2**

**23.3**

**Q2**

**28.3**

**Q2**

**31.8**

**Q2**

**23.3**

**Q2**

**21.6**

**Q3**

**76.7**

**Q3**

**71.7**

**Q3**

**68.2**

**Q3**

**76.7**

**Q3**

**78.4**

**Q3**

**84.4**

**Q3**

**83.6**

**Q3**

**85.2**

**Q3**

**86.0**

**Q3**

**86.3**

**Q2**

**13.7**

**Q2**

**14.0**

**Q2**

**14.8**

**Q2**

**16.4**

**Q2**

**15.6**

**Q2**

**4.5**

**Q3**

**95.5**

**Q1**

**0.0**

**Q4**

**0.0**

**Infected LRP6^(NKO)^**

**DKK1^(PKO)^ mice**

**B**

**Ly6G + ve cells (MFI)**

**C**

**D**

**Fig. S4: Impaired myeloperoxidase positive neutrophils obtained from LRP6^NKO^DKK1^(PKO)^ and LRP6^NKO^ infected mice.** Infected BALB/c, LRP6^NKO^ DKK1^PKO^ and LRP6^NKO^ mice were challenged with infective metacyclic promastigote (2 x 10^6^ parasites, n = 5) of WT strain via the footpad. Control mice (n = 10/2 feet per mouse) were given 0.9% NaCl saline. Cells from the infected footpad were collected on day 3 PI. Samples were analyzed by flow cytometry for myeloperoxidase-positive neutrophils. Representative dot plots indicate the percentage of myeloperoxidase-positive neutrophils **(A)**. The percentage of myeloperoxidase+ neutrophils obtained from each experimental group is indicated **(B)**. The MFI within the Ly6G+ cells and the number of neutrophils obtained from each experimental group are indicated **(C) & (D)**. In all the experiments, infected and non-infected BALB/c mice served as positive and negative controls, respectively.

**FSC-A**

**SSC-A**

**Ly6G-Pacific Blue**

**PI**

**SSC-A**

**Annexin V-FITC**

**A**

**Ly6G +ve**

**12.2**

**Q1**

**2.9**

**Q4**

**75.9**

**Q2**

**17.3**

**Q3**

**3.9**

**SSC-A**

**Ly6G**

**Annexin V-FITC**

**PI**

**Infected BALB/c**

**mice**

**Infected LRP6^(NKO)^**

**mice**

**Non-infected**

**mice**

**Infected LRP6^(NKO)^ DKK1^(PKO)^ mice**

**Q1**

**2.9**

**Q4**

**75.9**

**Q2**

**17.3**

**Q3**

**3.9**

**Q1**

**1.4**

**Q2**

**75.6**

**Q4**

**22.6**

**Q3**

**0.4**

**Q1**

**6.21**

**Q2**

**61.5**

**Q4**

**32.2**

**Q3**

**0.9**

**Q1**

**1.3**

**Q2**

**86.6**

**Q4**

**10.7**

**Q3**

**1.5**

**Ly6G +ve**

**12.2**

**Ly6G +ve**

**8.1**

**Ly6G +ve**

**5.0**

**Ly6G +ve**

**2.3**

**B**

**Fig. S5: Infected LRP6^NKO^ and LRP6^NKO^ DKK1^PKO^ mice manifest increased neutrophil apoptosis.** Infected BALB/c, LRP6^NKO^ DKK1^PKO^ and LRP6^NKO^ mice were challenged with infective metacyclic promastigote (2 x 10^6^ parasites, n = 5) of WT strain via the footpad. Control mice (n = 10/2 feet per mouse) were given 0.9% NaCl saline. Cells from the infected footpad were collected on day 3 PI. Samples were analyzed by flow cytometry for neutrophil apoptosis/viability. Representative dot plots indicate the percentage of apoptotic **(Q2 +Q3)** and viable **(Q4)** LY6G + cells obtained from concatenated samples of each experimental group **(A)** & **(B)**. Ly6G +ve cells indicate neutrophils. In all the experiments, infected and non-infected BALB/c mice served as positive and negative controls, respectively.

**A**

**SSC-A**

**FSC-A**

**Ly6G-PAC Blue**

**SSC-A**

**Annexin V-FITC**

**PI**

**Ly6G +ve**

**100.0**

**Q1**

**7.0**

**Q2**

**26.6**

**Q4**

**52.6**

**Q3**

**13.8**

**4-hr**

**0-hr**

**24-hr**

**Q1**

**7.0**

**Q2**

**26.6**

**Q4**

**52.6**

**Q3**

**13.8**

**Q1**

**2.9**

**Q2**

**20.0**

**Q3**

**11.5**

**Q4**

**65.7**

**Q1**

**2.2**

**Q2**

**14.5**

**Q3**

**11.5**

**Q4**

**71.8**

**Q1**

**2.0**

**Q2**

**8.3**

**Q4**

**80.3**

**Q3**

**9.4**

**Q2**

**7.5**

**Q3**

**8.6**

**Q4**

**82.0**

**Q1**

**1.9**

**Q1**

**0.0**

**Q2**

**2.4**

**Q3**

**3.7**

**Q4**

**93.9**

**Q4**

**94.0**

**Q4**

**91.9**

**Q4**

**80.4**

**Q4**

**79.1**

**Q1**

**1.6**

**Q1**

**1.8**

**Q1**

**0.8**

**Q1**

**0.0**

**Q3**

**3.0**

**Q2**

**3.0**

**Q3**

**4.5**

**Q2**

**2.8**

**Q3**

**9.2**

**Q3**

**10.8**

**Q2**

**8.5**

**Q2**

**8.6**

**Q2**

**0.6**

**Q3**

**3.9**

**Q4**

**95.4**

**Q1**

**0.1**

**Non-treated**

**10 ng /ml**

**rDKK1**

**100 ng/ml**

**rDKK1**

**30 ng/ml**

**rDKK1**

**50 ng/ml**

**rDKK1**

**FSC-A**

**Annexin V-FITC**

**Ly6G-PAC Blue**

**SSC-A**

**PI**

**SSC-A**

**Ly6G +ve**

**100.0**

**Ly6G +ve**

**100.0**

**Ly6G +ve**

**100.0**

**Ly6G +ve**

**100.0**

**Ly6G +ve**

**100.0**

**B**

**Ly6G +ve**

**100.0**

**Ly6G +ve**

**100.0**

**Fig. S6: Spontaneous neutrophil apoptosis was delayed by recombinant DKK1.** Neutrophils isolated from naïve mice as described in the Materials and Methods Section. Neutrophils were incubated with various concentrations of rDKK1. Neutrophil samples were harvested at 0-, 4-, and 24 hrs post incubation and were used to determine neutrophil apoptosis by flow cytometry. Representative contour plots indicate the percentage of total apoptotic Ly6G+ cells (Q2 +Q3) **(A)** & **(B)**. Ly6G +ve cells indicate neutrophils. In all the experiments, non-treated neutrophils served as controls.
